# Safety and Immunogenicity of the Malaria Vaccine Candidate MSP3 Long Synthetic Peptide in 12–24 Months-Old Burkinabe Children

**DOI:** 10.1371/journal.pone.0007549

**Published:** 2009-10-26

**Authors:** Sodiomon B. Sirima, Alfred B. Tiono, Alphonse Ouédraogo, Amidou Diarra, André Lin Ouédraogo, Jean Baptiste Yaro, Espérance Ouédraogo, Adama Gansané, Edith C. Bougouma, Amadou T. Konaté, Youssouf Kaboré, Abdoulaye Traoré, Chilengi Roma, Issiaka Soulama, Adrian J. F. Luty, Simon Cousens, Issa Nébié

**Affiliations:** 1 Centre National de Recherche et de Formation sur le Paludisme, Ouagadougou, Burkina Faso; 2 Groupe de Recherche et d'Action en Santé, Ouagadougou, Burkina Faso; 3 African Malaria Network Trust, Dar es Salaam, Tanzania; 4 Radboud University Nijmegen Medical Centre, Nijmegen, Netherlands; 5 London School of Hygiene and Tropical Medicine, London, United Kingdom; Prince Leopold Institute of Tropical Medicine, Belgium

## Abstract

**Background:**

A Phase Ia trial in European volunteers of the candidate vaccine merozoite surface protein 3 (MSP3), a *Plasmodium falciparum* blood stage membrane, showed that it induces biologically active antibodies able to achieve parasite killing *in vitro*, while a phase Ib trial in semi-immune adult volunteers in Burkina Faso confirmed that the vaccine was safe.

The aim of this study was to assess the safety and immunogenicity of this vaccine candidate in children aged 12–24 months living in malaria endemic area of Burkina Faso.

**Methods:**

The study was a double-blind, randomized, controlled, dose escalation phase Ib trial, designed to assess the safety, reactogenicity and immunogenicity of three doses of either 15 or 30 µg of MSP3-LSP adsorbed on aluminum hydroxide in 45 children 12 to 24 months of age randomized into three equal groups. Each group received 3 vaccine doses (on days 0, 28 and 56) of either 15 µg of MSP3-LSP, 30 µg of MSP3-LSP or of the Engerix B hepatitis B vaccine. Children were visited at home daily for the 6 days following each vaccination to solicit symptoms which might be related to vaccination. Serious adverse events occurring during the study period (1 year) were recorded. Antibody responses to MSP3-LSP were measured on days 0, 28, 56 and 84.

**Results:**

All 45 enrolled children received three MSP3 vaccine doses. No serious adverse events were reported. Most of the adverse events reported were mild to moderate in severity. The only reported local symptoms with grade 3 severity were swelling and induration, with an apparently dose related response. All grade 3 adverse events resolved without any sequelae. Both MSP3 doses regimens were able to elicit high levels of anti-MSP3 specific IgG1 and IgG3 antibodies in the volunteers with very little or no increase in IgG2, IgG4 and IgM classes: i.e. vaccination induced predominantly the isotypes involved in the monocyte-dependent mechanism of *P. falciparum* parasite-killing.

**Conclusion:**

Our results support the promise of MSP3-LSP as a malaria vaccine candidate, both in terms of tolerability and of immunogenicity. Further assessment of the efficacy of this vaccine is recommended.

**Trial Registration:**

ClinicalTrials.gov NCT00452088

## Introduction

The burden of malaria remains high in sub-Saharan countries despite the extensive deployment of existing control tools, such as insecticide-treated materials, intermittent preventive treatment, and artemisinin-based combination therapy (ACT) [1]. The development of an effective malaria vaccine could greatly contribute to disease control. In recent years, the effort to develop an effective malaria vaccine has resulted in a number of malaria vaccine candidates reaching the stage of testing in malaria-exposed populations. Most of the tested vaccines are either pre-erythrocytic or blood-stage malaria vaccine candidates.

One of the leading blood stage candidates is the merozoite surface protein 3 (MSP3), an antigen which is associated with the membrane of the free blood stage parasite. While most other vaccine candidates have been identified by experiments performed in experimental malaria models, MSP3 was identified by a clinical experiment in humans. The protection afforded by passive transfer of IgG from African adults into infected Thai children identified the cooperation of IgG with blood monocytes as a main defense mechanism in human beings in an antibody dependent, cellular inhibitory fashion (ADCI). Thereafter, the ADCI mechanism was used to screen a genome-wide expression library and identified MSP3 as the main target of antibodies mediating the monocyte-dependent *P. falciparum* killing effect. The monocyte-dependent mechanism implies that only the cytophilic classes of IgG, namely IgG1and IgG3, are important in mediating the effects, and epidemiological studies have confirmed that protection is associated with such cytophilic responses against MSP3 [2]–[5]. In malaria endemic areas, cytophilic anti-MSP3 and anti-crude *P.falciparum lysates* antibodies (IgG1 and IgG3) are dominant in clinically protected individuals, whereas non-cytophilic antibodies (IgG2 and IgM) predominate in clinically susceptible individuals [2], [6]–[8]. Finally, in contrast to most vaccine candidates and the N-terminal region of MSP3 [9], the C-terminal region of MSP3 is highly conserved from one parasite isolate to the other.

A long polypeptide chain (95 amino acids), derived from the highly conserved MSP3 C-terminal region, and produced in a single step by solid-phase synthesis (MSP3 long synthetic peptide (MSP3-LSP)), was tested in European, malaria-naïve volunteers and shown to be safe and immunogenic, inducing strong T-helper 1 and B-cell responses [10]. The induced antibodies were predominantly cytophilic and were able to inhibit *P. falciparum* erythrocytic growth in a monocyte dependent manner, under both *in vitro* and *in vivo* conditions [11]. A subsequent phase Ib trial in semi-immune adult volunteers, living in an area of Burkina Faso where malaria transmission is seasonally hyperendemic, found the vaccine to be safe at a dose of 30 µg, with less local reactogenicity in *P. falciparum* exposed individuals than was observed after the second and third doses in naïve volunteers [7]. This trial also suggested that the vaccine is able to stimulate cell-mediated immunity in individuals with some pre-existing immunity. However, no detectable humoral immune response was induced by the MSP3-LSP vaccine [12]. Although inducing a moderate cell-mediated immune response in adults with some pre-existing immunity, MSP3-LSP was well tolerated and met the preset GO criteria. Based on these findings, a phase 1b dose selection study was conducted in Burkina Faso to assess the safety, reactogenicity and immunogenicity of the vaccine in healthy children aged 1–2 years at enrolment.

## Materials and Methods

The protocol for this trial and supporting CONSORT checklist are available as supporting information; see Checklist S1 and Protocol S1.

### Ethic Statement

The trial protocol was approved by the national ethical committee of Burkina Faso and by the ethics committee of the London School of Hygiene and Tropical Medicine. The trial was conducted in compliance with principles set out by the International Conference on Harmonization Good Clinical Practices, the Declaration of Helsinki and the regulatory requirements of Burkina Faso.

Authorization to conduct the study was sought from the relevant administrative and health authorities in the study area. The assent of the community was obtained through a series of meetings with community opinion leaders and senior members. Individual written informed consent was obtained from all children's parents or legal representatives, in the presence of an impartial witness for illiterate parents/legal representatives.

The conduct of the study was monitored by the sponsor, African Malaria Network Trust (AMANET).

The study was overseen by a Data Safety Monitoring Board (DSMB), operating under a charter constituted by the sponsor. The DSMB and the local safety monitor reviewed the cumulative safety data and provided the investigators, through the sponsor, with a written authorization to proceed to the next vaccination and dose escalation at each stage.

### Study site

The trial was conducted at the Centre National de Recherche et Formation sur le Paludisme (CNRFP) malaria vaccine clinical trials center located in the village of Balonghin, in Saponé health district, 50 km southeast of Ouagadougou, the capital city of Burkina Faso. The area has been described elsewhere [12]–[13]. Malaria in this area is hyperendemic and transmission is seasonal. The climate is characteristic of the Sudanese savannah, with two distinct seasons: a dry season from November to May and a rainy season from June to October. The entomological inoculation rate in the area is estimated at less than one and more than 40 infective bites/person/month during the dry and rainy seasons, respectively. *P. falciparum* is the predominant malaria parasite, accounting for more than 95% of infections in children below the age of 5 during the high transmission season. The use of preventive measures (insecticide impregnated nets and indoor residual spraying) has been uncommon in the area.

The clinical trial center is equipped and staffed to undertake clinical trial phases 1b through 2b in compliance with national and international standards and requirements.

Saponé district hospital and the Pediatric Teaching Hospital in Ouagadougou both serve as referral hospitals for study participants requiring specialized care.

### Study design

The study was a double-blind, randomized, controlled, dose escalation phase Ib trial (www.clinicaltrials.gov NCT00452088) designed to assess the safety, reactogenicity and immunogenicity of three injections of either 15 µg or 30 µg of MSP3-LSP adsorbed on aluminum hydroxide in children 12 to 24 months of age. Trial participants were recruited among healthy children living in four villages within Saponé health district. The study duration was one year for enrolled participants.

### Screening and enrollment of study participants

A list of potentially eligible children was drawn from the Demographic Surveillance System (DSS) database run by the CNRFP since 2005 in the study area. The parents of these children were invited to the research center to discuss the trial, to obtain informed consent and for screening procedures if they consented to allow their child to participate in the study. Children were eligible for inclusion in the trial if they were found to be healthy after a general medical examination, if their parents indicated their intention to remain resident in the village for the study duration (12 months) and if their parents gave written informed consent. The first 45 children meeting the inclusion and exclusion criteria (Box S1) were enrolled, assigned a unique identification number and given an identity card to assist in correct identification.

### The study vaccines

The MSP3-LSP vaccine is a long synthetic peptide, produced by SYNPROSIS in France, and containing the amino-acid sequence 154–249 of the *P.falciparum* merozoite surface protein-3. It is 98.5% pure, very stable over time and underwent a full quality control process, including an assessment of potency, antigen content and conformity to specifications by HPLC and mass-spectrometry, one month before being employed for vaccination.

The vaccine was available in a multi-dose vial in lyophilized form. Prior to administration the vaccine was reconstituted in compliance with the Manufacturer's Standard Operating Procedure, and adjuvanted with aluminum hydroxide. Participants randomized to both MSP3 groups (MSP3-LSP 30 µg and MSP3-LSP 15 µg) received 0.5 mL of the reconstituted vaccine at each vaccination.

ENGERIX-B^®^ [Hepatitis B Vaccine (Recombinant)] vaccine is manufactured by GlaxoSmithKline Biologicals. It contains purified viral surface antigen expressed in *Saccharomyces cerevisiae* and contains no more than 5% yeast protein. ENGERIX-B^®^ was supplied as a sterile suspension, using the same adjuvant as MSP3-LSP (aluminum hydroxide), in a pre-filled syringe for intramuscular administration.

Each child in the comparator group received 0.5 mL of ENGERIX-B^®^ containing 10 µg of the hepatitis B surface antigen at each vaccination.

Both vaccines were administered by the subcutaneous route on days 0, 28, and 56 in alternating arms in the deltoid region. Immunizations were performed in 2007 from 26^th^ June to 11^th^ July (the beginning of malaria high transmission season) for the 1^st^ dose, 24^th^ July to 11^th^ August for the 2^nd^ dose and 21st August to 11^th^ September (the peak of malaria high transmission season) for the 3rd dose.

With Engerix B^®^, subcutaneous rather than intramuscular administration may result in lower anti-HBs antibodies GMT; however, a blood sample was taken at 6 months after the first dose to identify children who had not developed protective levels of antibodies. These were revaccinated with intramuscular injections at the same time as the MSP3-LSP recipients at the end of the study.

### Assessment of study endpoints

#### Safety and reactogenicity

At each vaccination visit, children underwent a physical examination before receiving the injection. Following each vaccination, the participants remained in the research center for at least one hour to assess immediate adverse events. They were then visited at home on each of the next 6 days after each dose to record any solicited or unsolicited local and systemic reactions. For the first dose, the last (day 6) evaluation was done at the research center.

Solicited local adverse events at the injection site included pain, swelling, induration, pruritus and erythema. Solicited systemic reactions included fever, loss of appetite, drowsiness and irritability/fussiness.

Any other symptoms not included in the targeted symptoms list above were recorded as non-solicited symptoms by the investigators.

An intensity grading scale was used to grade the severity of the adverse events. Severity of injection site reactions (other than pain and pruritus) was graded based on the measurement of the greatest surface diameter in mm. Grading was as follows: 0 =  absent, 1 = 0–5 mm, 2 = ≥5–20 mm, 3 = >20 mm. Axillary temperature was measured using a digital thermometer and fever grading was done as follows: 0 = <37.5°C, 1 = 37.5–38°C, 2 = 38.1°C–39°C, 3 = >39°C.

Pain, pruritus, and other solicited and unsolicited systemic reactions were graded as follows: 0 =  absent/none, 1 =  easily tolerated, 2 =  interferes with normal activity, 3 =  prevents normal daily activities.

Adverse events meeting one of the criteria for seriousness (death, life-threatening, requiring hospitalization) were reported as serious adverse events.

Venous blood samples were obtained at screening and on days 0, 6, 28, 56, and 84 to monitor the biological safety of the candidate vaccine. Hematological [hemoglobin, hematocrit, platelets, red blood cells (RBC) and white blood cells (WBC)], and biochemical [creatinine, total bilirubin, and the liver enzymes aspartate aminotransferase (ASAT) and alanine aminotransferase (ALAT)] parameters were assessed. The local laboratory references ranges for children aged from 12–24 months are presented in Box S2. A blood smear was prepared and a rapid diagnostic test for malaria was performed if a trial participant presented with an axillary temperature ≥37.5°C or history of fever within the last 24 hours. The rapid diagnostic test results were used to guide prompt treatment while awaiting the results of the slide examination. Malaria episodes were treated with Coartem^®^ according to the national guidelines for care in Burkina Faso.

All adverse events were followed up to resolution.

#### Immunological endpoints

Humoral immune responses on days 0, 28, 56, and 84 were evaluated using whole blood samples collected using heparin as an anticoagulant (VF-109SHL, Terumo Europe n.v.).

### MSP3-LSP and MSP3 overlapping Peptides

We studied immunological responses to the vaccine peptide MSP3-LSP which corresponds to a fully conserved region covering amino acids 181–276 (product number 00FS021#1B, Dictagene, Epalinges, CH) of the C-terminal region of MSP3 (386 amino acids long) from the *P. falciparum* strain Fc27 [14]. The sequence of the MSP3-LSP peptide is: RKTKEYAEKAKNAYEKAKNAYQKANQAVLKAKEASSYDYILGWEFGGGVP EHKKEENMLSHLYVSSKDKENISKENDDVLDEKEEEAEETEEEELE.

This peptide was synthesized, purified, bottled and lyophilized following good laboratory practices (GLP).

### Assessment of antibody responses

MSP3-LSP specific IgG, IgM and IgG1, IgG2, IgG3 and IgG4 subclass concentrations were measured by ELISA. The ELISA was done according to the Afro Immuno Assay standard operating procedure (SOP number AIA-007-03) [12]–[13]. In brief, microtiter plates (NUNC – Maxisorp F 96 439454)) were coated with the appropriate synthetic peptide (10 µg/ml concentration), incubated overnight at 4°C, and blocked with 3% milk powder in PBS-Tween 20 for 1 h. Plasma samples diluted 1∶200 (IgG and IgM) or 1∶25 (IgG subclasses) were added in duplicate and incubated at room temperature for 2 h. Plates were washed 4 times between each step. The antibody was detected using either peroxidase conjugated goat anti-human IgG or IgM (secondary antibody Caltag – H10007, H 15007). For IgG subclasses, the secondary antibody was a mouse anti-human monoclonal IgG subclass (clone NL16, Boehringer for IgG1, clone HP-6002 Sigma I-9513 for IgG2; Sky Bio, M08011, clone ZG4 for IgG3 and Sky Bio, M11014, clone RJ4 for IgG4). The antibody binding was revealed using peroxidase conjugated goat anti-mouse IgG. The subclass specific reagents used had been previously selected on the basis of low cross reactivities among themselves, and ability to faithfully react with African heavy chains dominant allotypes [15].

Bound secondary antibodies for IgG and IgM, and a third antibody group for IgG subclasses were quantified using ready-to-use TMB (3,3′, 5,5′-Tetramethylbenzidine) substrate. The optical density (OD) was read at 450 nm with a reference at 620 nm, and the OD value of the test-sample was converted into arbitrary units (AU) by means of a standard curve on each plate.

A pool of Burkinabe positive samples was used as a positive control and Danish plasma samples kindly provided by Michael Theisen from *Statens Serum Institute* (*Copenhagen, Denmark*) as negative controls.

### Randomization

The study participants were randomized to receive either MSP3-LSP at one of two doses (30 µg or 15 µg) or the control vaccine (Engerix B).

At the beginning of the study, the first group (22 children) was randomized to receive either 15 µg of MSP3-LSP (N = 15) or 10 µg of Engerix B (N = 7). Fourteen days after the first child from this group was immunized, a second group of 23 children was randomized to receive either 30 µg of MSP3-LSP (N = 15) or Engerix B (N = 8). Thus 15 children were randomized to each study arm.

Randomization codes were independently created by the study statistician and participants were randomly assigned treatment numbers upon presentation for the first dose of vaccine.

A copy of the randomization list was given to the pharmacist in charge of the vaccine preparation and to the local safety monitor appointed by the sponsor.

### Blinding

The study was a double blind trial with the vaccine recipient and their parent(s)/guardian(s), as well as those responsible for the evaluation of safety and immunogenicity endpoints, unaware of the treatment assignment of each study participant until the end of the study. The only study personnel aware of the vaccine assignment were those responsible for the storage and preparation of vaccines, and they did not play any other role in the study.

Since the appearance of the two vaccines was different, opaque tape was placed over the syringes to prevent the investigators from seeing their contents.

Unblinding of the investigators occurred once the study data up to day 84 had been entered and the database locked.

### Statistical methods

The sample size was based on the safety endpoints. With 14 children completing follow-up in each MSP3 arm, (15 µg and 30 µg) the study had 90% power to detect at least one MSP3 vaccinated individual with a systemic reaction (or a serious adverse event) if the underlying risk of such an event was 15% or more. One additional subject per arm was included to ensure adequate sample size in case of dropout for any reason. Thus, 45 participants in total were recruited (15 receiving MSP-LSP 15 µg, 15 receiving MSP-LSP 30 µg, 15 receiving Engerix B^®^).

A statistical analysis plan was established for the trial. All original data were transcribed from source documents to case report forms which were counterchecked before computer entry. Descriptive analyses were performed using STATA version 10 (College Station, TX, USA). Given the multiplicity of endpoints considered and the small sample size, formal statistical tests were not used to compare vaccine groups. Geometric mean concentrations of anti-MSP3-LSP antibodies and 95% confidence intervals were calculated.

## Results

Based on the age criterion, 110 children were invited for screening, 59 of whom were eligible. Of these, the first 45 children coming on the day of vaccination were enrolled. Common reasons for exclusion included moderate malnutrition (n = 27) and the following biological abnormalities (n = 16): anemia, high value of ASAT and leukocytosis. Fourteen eligible subjects were not enrolled since the target sample size had been achieved.

**Figure 1:**
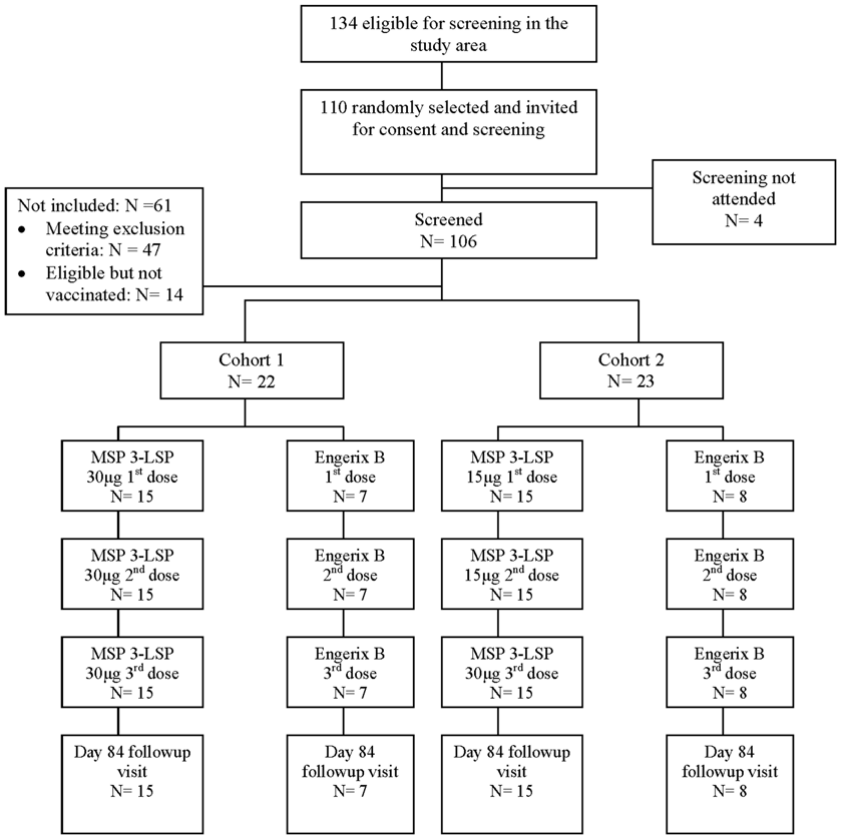
Trial profile.

All participants received three doses of their allotted vaccine and completed follow up to day 84. No dropouts were recorded and there were no withdrawals due to vaccine adverse effects (Figure 1).

The baseline characteristics of the three vaccine groups were broadly similar (Table 1).

**Table 1:** Baseline characteristics of the study participants at enrollment within each vaccine group.

Characteristics	MSP3 (15 µg) (mean±SD)	MSP3 (30 µg) (mean±SD)	Engerix B (mean±SD)
**Number randomized**	15	15	15
**Age (months)**	18.8±3.6	17.4±3.2	18.0±3.4
**Weight (Kg)**	10.0±1.4	9.3±1.0	9.0±0.9
**Height (Cm)**	79.7±4.8	77.1±3.9	77.5±4.4
**RBC (10^6^/µL)**	4.8±0.7	4.7±0.7	4. 6±0.7
**WBC (10^3^/µL)**	11.3±2.2	10.7±2.6	11.0±1.8
**Hemoglobin (g/dL)**	9.8±1.0	10.1±1.1	10.1±1.1
**Hematocrit (%)**	30.9±2.7	31.5±3.3	31.7±2.9
**Platelets (10^3^/µL)**	404.5±173.7	323.9±135.8	322.2±128.7
**Creatinine (µmol/L)**	40.1±6.1	39.5±6.2	39.4±5.9
**ASAT (U/L)**	59.1±10.7	53.3±16.6	52.1±21.8
**ALAT (U/L)**	32.7±6.14	37.8±9.0	40.8±9.6
**Total bilirubin (µmol/L)**	7.4±3.6	6.8±2.5	7.1±3.7

### Safety

The vaccines were well tolerated. No serious adverse events were seen. The incidence of solicited injection site reactions within the 7 day follow up period after each vaccination is reported in Table 2. The most common local reactions were pain, swelling and induration. Children in both MSP3-LSP groups experienced more local symptoms than those who received the control vaccine. Overall, local reactions were no more common in the MSP3-LSP 30 µg than in the MSP3-LSP 15 µg group. Symptoms of grade 3 severity were only reported for swelling and induration and were more common in the MSP3-LSP vaccine group after the second and third vaccinations (Table 2). After the second vaccination most of the volunteers (9/15) in the 30 µg group experienced grade 3 severity swelling, while only 1 volunteer (1/15) in each of 15 µg and Engerix groups had a similar reaction. At the third dose 7 and 6 children respectively in the MSP3 groups (30 and 15 µg) had grade 3 swelling compared to 2 children in the Engerix group. The majority of the MSP3-LSP 30 µg group had grade 3 induration at the injection site after the second (11/15) and the third (14/15) doses. Grade 3 induration events were less common in the MSP3-LSP (15 µg) and Engerix groups. All grade 3 adverse events resolved without any sequelae.

**Table 2:** Incidence of injection site solicited adverse events according to vaccine groups.

		Dose 1			Dose 2			Dose 3		
Events	Intensity	MSP3 (15 µg) N = 15	MSP3 (30 µg) N = 15	Engerix B N = 15	MSP3 (15 µg) N = 15	MSP3 (30 µg) N = 15	Engerix B N = 15	MSP3 (15 µg) N = 15	MSP3 (30 µg) N = 15	Engerix B N = 15
**Pain**										
	Any	**12**	**12**	**2**	**9**	**8**	**2**	**6**	**7**	**1**
	Grade 1	**12**	**10**	**2**	**8**	**8**	**2**	**6**	**7**	**1**
	Grade 2	0	**2**	0	**1**	0	0	0	0	0
	Grade 3	0	0	0	0	0	0	0	0	0
**Swelling**										
	Any	**14**	**11**	**5**	**5**	**10**	**2**	**11**	**8**	**3**
	Grade 1	0	**1**	0	0	0	0	0	0	0
	Grade 2	**9**	**8**	**4**	**4**	**1**	**1**	**5**	**1**	**1**
	Grade 3	**5**	**3**	**1**	**1**	**9**	**1**	**6**	**7**	**2**
**Induration**										
	Any	**13**	**14**	**8**	**15**	**12**	**7**	**15**	**15**	**10**
	Grade 1	0	0	0	0	0	0	0	0	0
	Grade 2	**11**	**13**	**8**	**10**	**1**	**6**	**6**	**1**	**5**
	Grade 3	**2**	**1**	0	**5**	**11**	**1**	**9**	**14**	**5**
**Erythema**										
	Any	0	**1**	**1**	0	0	**2**	0	0	**1**
	Grade 1	0	**1**	0	0	0	**1**	0	0	0
	Grade 2	0	0	**1**	0	0	**1**	0	0	**1**
	Grade 3	0	0	0	0	0	0	0	0	0

Table 3 presents the incidence of general solicited symptoms. The most frequently reported solicited symptom was mild drowsiness, reported in all three vaccine groups. The only occurrences of grade 3 solicited general symptoms were 5 cases of fever greater than 39°C. None were judged to be related to the study vaccines. Two cases of grade 3 fever were reported in the MSP3-LSP 30 µg group after the second vaccine dose, one of which was a malaria episode occurring on Day 6, while the second case was associated with a gastroenteritis episode on Day 5. In the Engerix B group, three cases were recorded, all due to malaria.

**Table 3:** Incidence of general solicited adverse events according to vaccine groups.

		Dose 1			Dose 2			Dose 3			
Events	Intensity	MSP3 (15 µg) N = 15	MSP3 (30 µg) N = 15	Engerix B N = 15	MSP3 (15 µg) N = 15	MSP3 (30 µg) N = 15	Engerix B N = 15		MSP3 (15 µg) N = 15	MSP3 (30 µg) N = 15	Engerix B N = 15
**Fever**											
	**Any**	0	**2**	**2**	**1**	**3**	**5**		0	**4**	**2**
	**Grade 1**	0	0	0	**1**	**1**	**2**		0	**3**	**1**
	**Grade 2**	0	**2**	**1**	0	0	**1**		0	**1**	**1**
	**Grade 3**	0	0	1	0	2	2		0	0	0
	**Grade 3 related**	0	0	0	0	0	0		0	0	0
**Irritability**											
	**Any**	0	**1**	0	**1**	0	0		0	**3**	0
	**Grade 1**	0	**1**	0	**1**	0	0		0	**3**	0
	**Grade 2**	0	0	0	0	0	0		0	0	0
	**Grade 3**	0	0	0	0	0	0		0	0	0
	**Grade 3 related**	0	0	0	0	0	0		0	0	0
**Drowsiness**											
	**Any**	0	**5**	**1**	**5**	**3**	**1**		**2**	**4**	**5**
	**Grade 1**	0	**5**	0	**5**	**3**	1		**2**	**4**	**5**
	**Grade 2**	0	0	0	0	0	0		0	0	0
	**Grade 3**	0	0	0	0	0	0		0	0	0
	**Grade 3 related**	0	0	0	0	0	0		0	0	0
**Loss of appetite**											
	**Any**	0	**1**	0	0	0	0		0	0	0
	**Grade 1**	0	0	0	0	0	0		0	0	0
	**Grade 2**	0	0	0	0	0	0		0	0	0
	**Grade 3**	0	0	0	0	0	0		0	0	0
	**Grade 3 related**	0	0	0	0	0	0		0	0	0

One participant in the MSP3-LSP 30 µg group experienced a grade 3 unsolicited adverse events (hepatitis A) after the 2nd immunization (Table 4). The event was not judged to be related to the vaccine.

**Table 4:** Incidence of unsolicited adverse events according to vaccine groups.

Events	Intensity	MSP3 (15 µg) N = 15	MSP3 (30 µg) N = 15	EngerixB N = 15
Uncomplicated malaria a	Any	35	26	35
	Grade 1	30	23	30
	Grade 2	5	3	5
	Grade 3	0	0	0
Respiratory Infection	Any	16	19	15
	Grade 1	15	14	13
	Grade 2	1	5	2
	Grade 3	0	0	0
Diarrhea	Any	3	5	1
	Grade 1	3	4	1
	Grade 2	0	1	0
	Grade 3	0	0	0
Wound/dermatosis	Any	9	3	4
	Grade 1	7	2	2
	Grade 2	2	1	2
	Grade 3	0	0	0
Hepatitis A	Any	0	2	1
	Grade 1	0	1	1
	Grade 2	0	0	0
	Grade 3	0	1	0
Others^b^	Any	3	5	1
	Grade 1	2	3	0
	Grade 2	1	2	1
	Grade 3	0	0	0

a malaria was define as fever (temperature > = 37.5) + any asexual parasitemia in absence of any evidence cause of fever recorded since the administration of the first dose of the vaccine.

b Others MSP3 (15 µg):Intoxication with oil, Stomatitis, splenomegaly; Others MSP3 (30 µg):Intoxication with oil, conjonctivis, fever without etiology.

Others EngerixB: fever without etiology.

Biological safety data did not show any important differences in the numbers of participants with out-of-range values in the three vaccine groups (Table 5). None of the out-of-range values were judged to be related to vaccination, and none were considered clinically important.

**Table 5:** Number of children with abnormal biological values after each vaccination^a^.

Parameters	Time point of assessment	MSP3 (15 µg) N = 15	MSP3 (30 µg) N = 15	Engerix B N = 15
**RBC**				
	6 days post dose 1	6	5	5
	1 month post dose 1	5	6	5
	1 month post dose 2	7	6	6
	1 month post dose 3	5	3	4
**WBC**				
	6 days post dose 1	0	0	1
	1 month post dose 1	2	1	1
	1 month post dose 2	0	1	0
	1 month post dose 3	0	1	0
**Hb**				
	6 days post dose 1	0	0	0
	1 month post dose 1	1	0	0
	1 month post dose 2	0	1	0
	1 month post dose 3	1	2	1
**Platelets**				
	6 days post dose 1	0	1	0
	1 month post dose 1	0	0	0
	1 month post dose 2	0	0	0
	1 month post dose 3	0	0	0
**Creatinine**				
	6 days post dose 1	0	0	0
	1 month post dose 1	0	0	0
	1 month post dose 2	0	0	0
	1 month post dose 3	0	0	0
**ASAT**				
	6 days post dose 1	8	6	8
	1 month post dose 1	10	12	12
	1 month post dose 2	3	5	9
	1 month post dose 3	6	5	6
**ALAT**				
	6 days post dose 1	1	0	1
	1 month post dose 1	0	2	0
	1 month post dose 2	0	0	2
	1 month post dose 3	0	1	0
**Total Bilirubin**				
	6 days post dose 1	3	3	6
	1 month post dose 1	2	2	1
	1 month post dose 2	1	5	2
	1 month post dose 3	0	1	1

a All observed abnormal values were mild and not clinically significant.

### Humoral immune responses

**Figure 2:**
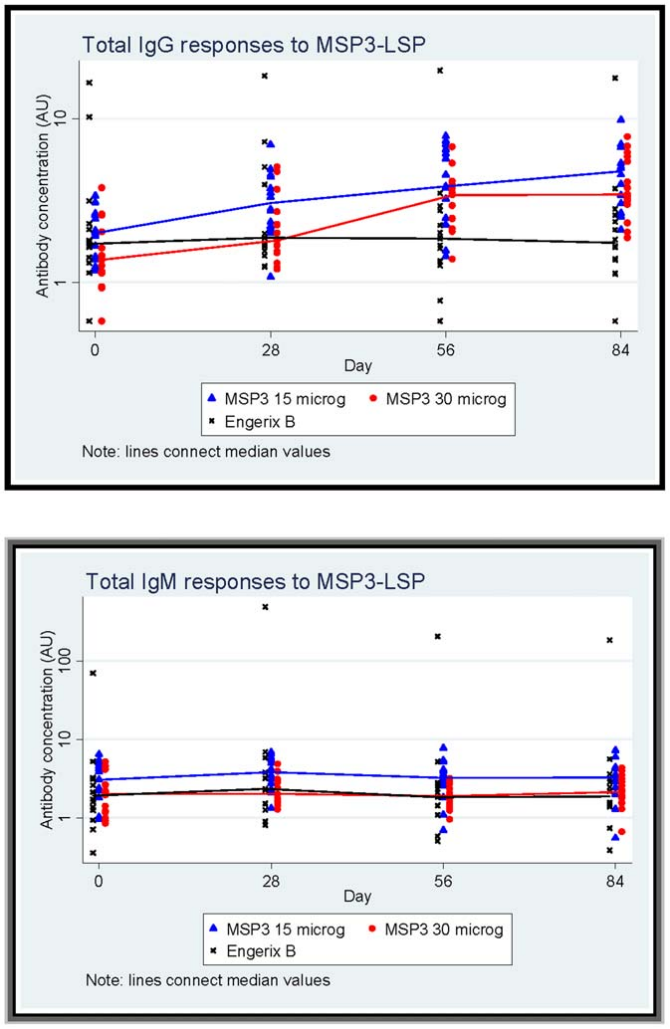
Total IgG and IgM antibody responses to MSP3-LSP by vaccine group. Symbols represent individual value of antibody measured by ELISA in a vaccine group and continuous line connects median values of arbitrary units of antibody responses to MSP3-LSP at different time points by vaccine groups: ▴ Full triangles and line in blue for MSP3-LSP (15 µg) group. • Full circles and line in red for MSP3-LSP (30 µg) group. x Cross symbols and line in black for Engerix group.

**Figure 3:**
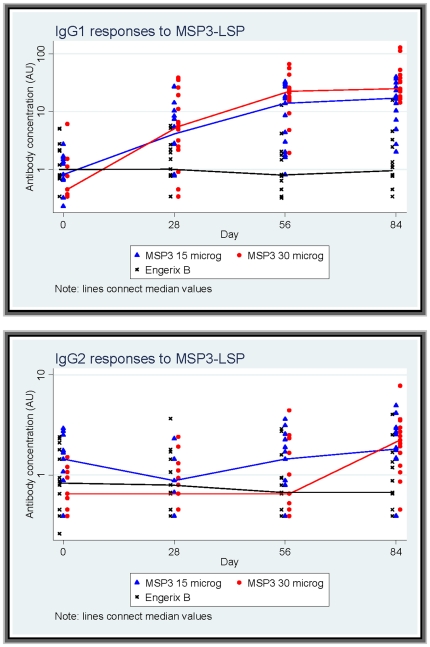
Antibody responses of IgG subclasses to MSP3-LSP by vaccine group. Symbols represent individual value of antibody measured by ELISA in a vaccine group and continuous line connects median values of arbitrary units of antibody responses to MSP3-LSP at different time points by vaccine groups: ▴ Full triangles and line in blue for MSP3-LSP (15 µg) group. • Full circles and line in red for MSP3-LSP (30 µg) group. x Cross symbols and line in black for Engerix group.

Humoral immune response results are summarized in Figures 2 and 3. Total IgG, IgM and IgG1, IgG2, IgG3 and IgG4 subclasses were measured at baseline (D0) and at one month intervals after each vaccination (D28, D56 and D84). At baseline, the levels of IgG antibody responses to MSP3-LSP were broadly similar in the three groups. The total IgG response to MSP3-LSP appeared to increase in both MSP3-LSP vaccine groups following vaccination while no increase in anti-MSP3 antibody was observed in children receiving the control (hepatitis B) vaccine. No marked changes in antibody responses of the IgM class were seen in any of the vaccine groups (Figure 2).

The cytophilic IgG (IgG1 and IgG3) and the non-cytophilic IgG (IgG2 and IgG4) responses to MSP3-LSP are presented in Figure 3. In both MSP3-LSP vaccine groups, cytophilic antibody responses (IgG1 and IgG3) were markedly higher post-vaccination than at baseline. In the control group (Engerix B), cytophilic IgG antibody responses were similar pre- and post-vaccination. No changes in IgG2 and IgG4 responses were evident in the control arm. Responses post-vaccination appeared to have increased by Day 84 in the MSP3-LSP 30 µg group, but these increases were much less marked than those observed for IgG1 and IgG3 subclasses. In the 15 µg group any increases in IgG2 or IgG4 responses were small enough to be compatible with random variation.

## Discussion

This study was designed to evaluate the reactogenicity, safety and immunogenicity of *P. falciparum* Merozoite Surface Protein-3 Long Synthetic Peptide (MSP 3-LSP) with aluminum hydroxide as adjuvant in 12 to 24 month old children in Burkina Faso. The study provides evidence of the safety and tolerability of MSP3-LSP when given to young children with prior exposure to malaria in Burkina Faso. We opted for a dose escalating design because this was the first time this vaccine had been given to young children.

In comparison to the previous trials, MSP3-LSP appears to be more reactogenic at the site of injection than the comparator Engerix-B vaccine, with a higher proportion of MSP3-LSP recipients presenting grade 3 local reactions (swelling and induration) [3–4;16–17]. With the exception of localized symptoms at the site of injection which all resolved spontaneously in a short time, post-vaccination follow up recorded a similar incidence and intensity of general and unsolicited symptoms among those receiving MSP3-LSP and those receiving the Engerix B vaccine. The proportion of children who had biological values outside the normal ranges for the measured parameters was similar in all vaccine groups. These findings are consistent with previous trials carried out in the same area in semi-immune adults which showed MSP3-LSP with aluminum hydroxide to be safe and well-tolerated [12].

Humoral immune responses (IgG, IgM and IgG subclasses (IgG1, IgG2, IgG3 and IgG4)) were investigated at baseline (D0) and at one month intervals after each dose (D28, D56 and D84). The MSP3-LSP vaccine induced IgG antibody responses of which the strongest were of cytophilic antibody sub-classes (IgG1 and IgG3). These two classes of cytophilic antibodies have been reported to play a key role both *in vitro* and *in vivo* in the monocyte mediated antibody-dependent inhibition of parasite growth known as antibody dependent cellular inhibition (ADCI) [3–4;16–17]. The two cytophilic classes IgG1 and IgG3 induced in our study are the only ones able to bind by their heavy chains to the Fc-gamma receptors RIIa and RIIIa to induce the antiparasite, monocyte-dependent, ADCI effect [18].

These cytophilic antibody responses, particularly anti-MSP3 IgG3, have been shown to be strongly associated with acquired clinical protection in humans, [3–4;19–22]. Similar results were seen in malaria naïve, immunized Swiss volunteers where the MSP3-LSP vaccine also generated cytophilic antibodies [10].

Non-cytophilic IgG (IgG2 and IgG4) antibody responses to vaccination with MSP3-LSP were much less marked. The very low levels or absence of non-cytophilic IgG2, IgG4 and IgM classes is a very positive finding. Non-cytophilic antibodies could compete for the same antigenic target as that used by ADCI which may inhibit the bridging of merozoites to human monocytes by cytophilic antibodies. If this had occurred it could potentially reduce the ability of cytophilic antibodies to control parasite multiplication by the ADCI mechanism [20].

Humoral immune responses in young children with limited exposure to natural *P. falciparum* contrast with those of our previous trial conducted in adults. In the previous trial, no increase in anti-MSP3 antibody concentrations was seen, probably because the subjects already had high levels of pre-existing immunity resulting from prior long standing natural exposure [12].

The rapid increase in antibodies after vaccination may indicate that, although very young, the children have been primed by the parasite antigen prior to vaccination. However, the generally low level of anti-MSP3 specific responses in the control Engerix group also suggests low overall exposure to malarial antigens in general, and to MSP3 antigens in particular in the cohort of children vaccinated.

### Conclusion

These results demonstrate the short- and medium-term safety and immunogenicity of the MSP3-LSP vaccine in children living in an area characterized by stable seasonal malaria transmission in Burkina Faso. The safety of both dose levels of MSP3 was acceptable. However, a lower incidence of local reactogenicity was observed with the control vaccine. The immunogenicity of MSP3-LSP was demonstrated by the higher IgG responses observed in both dose groups compared both to baseline levels and to the levels in the group given the comparator vaccine. The profile of the induced antibody responses favored the cytophilic subclasses (IgG1 and IgG3), and the higher dose of 30 µg appeared to induce stronger responses that the 15 µg dose.

Given our results, we believe that the 30 µg dose of MSP3 should be evaluated in larger trials to investigate both immunogenicity and *in vivo* biological activity as demonstrated by, for example, protection against malaria episodes or reduced severity of malarial disease.

## Supporting Information

Box S1 (0.02 MB RTF)

Box S2 (0.02 MB RTF)

Protocol S1 Trial Protocol
(0.95 MB DOC)

Checklist S1 CONSORT Checklist
(0.07 MB RTF)
