# Correction: Safety and Immunogenicity of the Malaria Vaccine Candidate MSP3 Long Synthetic Peptide in 12–24 Months-Old Burkinabe Children

**DOI:** 10.1371/annotation/3221b8d9-038d-4e67-82fa-de6b1233b459

**Published:** 2010-04-29

**Authors:** Sodiomon B. Sirima, Alfred B. Tiono, Alphonse Ouédraogo, Amidou Diarra, André Lin Ouédraogo, Jean Baptiste Yaro, Espérance Ouédraogo, Adama Gansané, Edith C. Bougouma, Amadou T. Konaté, Youssouf Kaboré, Abdoulaye Traoré, Chilengi Roma, Issiaka Soulama, Adrian J. F. Luty, Pierre Druilhe, Simon Cousens, Issa Nébié

In our recent article, we failed to include Prof Pierre Druilhe as an author. Pierre Druilhe contributed to the design and implementation of the trial, the writing of the protocol, the interpretation of the results, and the preparation of the article, and thus should have been listed as a contributor on this study. We apologise for this omission and would like to correct the author list to the following:

Sirima SB, Tiono AB, Ouédraogo A, Diarra A, Ouédraogo AL, Yaro JB, Ouédraogo E, Gansané A, Bougouma EC, Konaté AT, Kaboré Y, Traoré A, Chilengi R, Soulama I, Luty AJ, Druilhe P, Cousens S, Nébié I.

The revised author list also includes the correct citation for Dr Chilengi as 'Chilengi R'.

We would also like to clarify that the sponsor African Malaria Network Trust requested and was shown a copy of the manuscript before it was submitted for publication.

