# Supplementary material for: Safety and Immunogenicity of the Malaria Vaccine Candidate MSP3 Long Synthetic Peptide in 12–24 Months-Old Burkinabe Children
Source: PLoS One. 2009 Oct 26;4(10):e7549. doi: 10.1371/journal.pone.0007549 (PMC2764341; doi:10.1371/journal.pone.0007549)
Supplement: Box S1 — (0.02 MB RTF) [file pone.0007549.s001.rtf]

1.	Box 1 : Exclusion criteria 


·	Symptoms and/or physical signs of disease that could interfere with the interpretation of the trial results or compromise the health of the participant; 
·	Immunosuppressive therapy (steroids, immune modulators or immune suppressors) within 3 months prior to recruitment; 
·	Any social, psychological or geographical issues that could prevent the follow up; 
·	Use of any investigational drug or vaccine other than the study vaccine within 30 days preceding the first dose of the study vaccine, or planned use up to 30 days after the third dose; 
·	Suspected or known hypersensitivity to any of the vaccine components or to a previous vaccine; 
·	Any clinically significant abnormal/out of range results on screened laboratory blood samples; 
·	Planned administration of a vaccine not foreseen by the study protocol within 30 days before the first dose of vaccine; an exception is the receipt of an Expanded Program on Immunization (EPI) or licensed vaccine (measles, oral polio, meningococcal and combined diphtheria/pertussis/tetanus vaccines) which may be given 14 days or more before or after vaccination; 
·	evidence of chronic or active hepatitis B infection; 
·	Presence of chronic illness that, in the judgment of the investigator, would interfere with the study outcomes or pose a threat to the participant's health; 
·	Administration of immunoglobulin and/or any blood products within the three months preceding the first dose of study vaccine or planned administration during the study period;
·	 History of surgical splenectomy; xii) moderate malnutrition at screening defined as weight for age Z-score of less than -2. 	
