# Supplementary material for: Safety and Immunogenicity of the Malaria Vaccine Candidate MSP3 Long Synthetic Peptide in 12–24 Months-Old Burkinabe Children
Source: PLoS One. 2009 Oct 26;4(10):e7549. doi: 10.1371/journal.pone.0007549 (PMC2764341; doi:10.1371/journal.pone.0007549)
Supplement: Box S2 — (0.02 MB RTF) [file pone.0007549.s002.rtf]

Box 2: Local laboratory references ranges for children aged from 12 – 24 months


Analysis
Unit 
Low Reference Value 
High Reference Value 


Serum creatinine 
µmol/L
0
60

Serum total bilirubin 
µmol/L
3.42
20.52

Serum aspartate aminotransferase (AST) 
UI/L (37°C)
0
40

Serum alanine aminotransferase (ALT) 
UI/L (37°C)
0
60

WBC count
103/µL
4
17

RBC count 
106/µL
3.7
5.0

Platelet count
103/µL
100
-

Haemoglobin 
g/dL
8.0
-

	
