# Supplementary material for: Safety and Immunogenicity of the Malaria Vaccine Candidate MSP3 Long Synthetic Peptide in 12–24 Months-Old Burkinabe Children
Source: PLoS One. 2009 Oct 26;4(10):e7549. doi: 10.1371/journal.pone.0007549 (PMC2764341; doi:10.1371/journal.pone.0007549)
Supplement: Protocol S1 — Trial Protocol (0.95 MB DOC) [file pone.0007549.s003.doc]

**TRIAL PROTOCOL**

| A double blind, randomized, controlled, dose escalation phase Ib field trial in 12 to 24 month old children in Burkina Faso to evaluate the safety and immunogenicity of the *Plasmodium falciparum* Merozoite Surface Protein-3 Long synthetic peptide (MSP 3) adjuvanted in aluminium hydroxide versus hepatitis B |
| --- |

| **TRIAL IDENTIFIER** | | **MSP3_BF_0302** |
| --- | --- | --- |
| **Product** | | **MSP3-LSP** |
|  | *Form* | lyophilised, adsorbed in Aluminium Hydroxide |
|  | *Route* | sub-cutaneous |
|  | *Dosage* | 15µg and 30µg of MSP3 |
|  |  |  |
| **Trial Centre:** | | **Projet de Développement de Vaccins Anti-Paludique- Centre National de Recherche et de Formation sur le Paludisme**  **(PDVAP/CNRFP) –** |
|  |  | 01 BP 2208 Ouagadougou 01  Burkina Faso  Tel: + 226 50 32 46 95/6  Fax: + 226 50 30 52 20 or + 226 50 31 04 77 |
| **Principal Investigator** | | **Sodiomon Bienvenu SIRIMA, BA,** MD, PhD (CNRFP) |
|  |  |  |
| **Investigator(s)** | | **Tiono Alfred,** MD (CNRFP) |
|  |  | **Amadou T. KONATE,** MD (CNRFP) |
|  | | **Alphonse OUEDRAOGO,** MD (CNRFP) |
|  | | **Adama GANSANE,** Pharm D, (CNRFP) |
|  | | **Abdoulaye TRAORE**, MA, PhD (CNRFP) |
|  | |  |
| **Biological Evaluator(s)** | | **Issa NEBIE**, MSc, PhD (CNRFP) |
|  | | **Amidou DIARRA**, MSc (CNRFP) |
|  | |  |
| **Inventor:** | | **Pierre DRUILHE,** MD, PhD **(**Institut Pasteur Paris) |
|  | |  |
| **Sponsor** | | **African Malaria Network Trust (AMANET)**  **Roma Chilengi**, BSc. MB ChB, Clin Research, DHTM  Clinical Trials Coordinator,  Tanzania Commission for Science and Technology Building Building,  P.O. Box 33207  Dar es salaam, Tanzania  Tel: (255) 22 2700018 Fax:  255 (0)22 2700380  E-mail: [chilengi@amanet-trust.org](mailto:chilengi@amanet-trust.org)  Web-site:   [www.amanet-trust.org](http://www.amanet-trust.org/) |

| **Immunology Quality Assurance** | **Adrian Luty PhD**  Medical Parasitology-268 MMB-NCMLS.  Radboug University Nijmegen Medical Centre,  PO Box 9101, 6500 HB Nijmegen  Netherlands  Tel. 31-(0)24-3613663  Fax. 31-(0)24-3614666  e-mail [a.luty@mmb.umcn.nl](mailto:a.luty@mmb.umcn.nl) |
| --- | --- |
|  |  |
| **Laboratory Quality Control** | **John Waitumbi DVM, PhD**  Laboratory Director and Senior Scientist  KEMRI/WRP  Tel: +254 57 2022942  e-mail: jwaitumbi@wrp-ksm.org |
|  |  |
| **Clinical Monitor** | **Mahamadou Aly THERA,** MD, MPH  Malaria Research and Training Center (MRTC)  Department of Epidemiology of Parasitic Diseases (DEAP) Faculty of Medicine, Pharmacy and Dentistry, (FMPOS)  University of Mali; BP: 1805 Bamako |
|  |  |
| **Statistician** | **Simon COUSENS,** PhD  London School of Hygiene and Tropical Medicine Keppel Street London WC1E 7HT  Tel: (44) 20 7927 2422 , Fax: (44) 20 7636 8739  e-mail: [simon.cousens@lshtm.ac.uk](mailto:simon.cousens@lshtm.ac.uk) |
|  |  |
| **Vaccine Quality Assurance** | **Jean-Pierre Salles**  13382 Marseille Cedx 13  France  Tel: +33 491 118800  e-mail: jpsalles@synprosis.com |
|  |  |
| **Data Safety Monitoring Board** | **TBA** |
|  |  |
| **Version** | Third draft |
|  |  |
| **Date** | January 2007 |
|  |  |

[Study phases 9](#__RefHeading___Toc222918674)

[Clinic Visit 9](#__RefHeading___Toc222918675)

[1. Background Information 11](#__RefHeading___Toc222918676)

[1.1. Introduction 11](#__RefHeading___Toc222918677)

[1.2. Epidemiology 12](#__RefHeading___Toc222918678)

[1.3. Name and description of investigational product 13](#__RefHeading___Toc222918679)

[1.4. Summary of findings from non-clinical studies 13](#__RefHeading___Toc222918680)

[1.5. Clinical experience with MSP 3 LSP 15](#__RefHeading___Toc222918681)

[1.6. Rationale 17](#__RefHeading___Toc222918682)

[2. Trial Objectives 18](#__RefHeading___Toc222918683)

[2.1. Primary Objective 18](#__RefHeading___Toc222918684)

[2.2. Secondary Objectives 18](#__RefHeading___Toc222918685)

[2.3. Tertiary or exploratory objectives 18](#__RefHeading___Toc222918686)

[3. Study Design Overview 19](#__RefHeading___Toc222918687)

[4. Trial design and methodology 20](#__RefHeading___Toc222918688)

[4.1. Description and justification of design 20](#__RefHeading___Toc222918689)

[4.2. Study Site 21](#__RefHeading___Toc222918690)

[5. Conduct of the trial 21](#__RefHeading___Toc222918691)

[5.1. Recruitment procedure 21](#__RefHeading___Toc222918692)

[5.2. Randomisation and treatment allocation 21](#__RefHeading___Toc222918693)

[5.2.1. Randomisation of participants 21](#__RefHeading___Toc222918694)

[5.2.2. Method of blinding and breaking the study blind 22](#__RefHeading___Toc222918695)

[5.2.3. Trial Calendar 22](#__RefHeading___Toc222918696)

[5.2.4. Vaccination and serological schedule 22](#__RefHeading___Toc222918697)

[5.2.5. Clinical evaluation and follow up 23](#__RefHeading___Toc222918698)

[5.2.6. Safety monitoring plan 23](#__RefHeading___Toc222918699)

[5.2.7. Data Safety Monitoring Board (DSMB) 23](#__RefHeading___Toc222918700)

[5.2.8. Local Safety Monitor (LSM) 24](#__RefHeading___Toc222918701)

[5.2.9. Data Reviewed by the LSM 24](#__RefHeading___Toc222918702)

[5.2.10. Safety monitoring reports 24](#__RefHeading___Toc222918703)

[5.2.11. Process for the suspension of progression to the next sequential vaccine dose or to the dose escalation 25](#__RefHeading___Toc222918704)

[5.2.12. Process if the trial is suspended on the basis of safety reports 25](#__RefHeading___Toc222918705)

[5.3. Termination of the trial 26](#__RefHeading___Toc222918706)

[6. Data collection and handling 26](#__RefHeading___Toc222918707)

[6.1. Source data 26](#__RefHeading___Toc222918708)

[6.2. Case Report Forms 26](#__RefHeading___Toc222918709)

[6.3. Data processing 27](#__RefHeading___Toc222918710)

[7. Handling Biological samples 27](#__RefHeading___Toc222918711)

[7.1. Immunological samples 27](#__RefHeading___Toc222918712)

[7.2. Storage Conditions 28](#__RefHeading___Toc222918713)

[7.3. Biological evaluation samples 28](#__RefHeading___Toc222918714)

[8. Trial participants 29](#__RefHeading___Toc222918715)

[8.1. Recruitment of participants 29](#__RefHeading___Toc222918716)

[8.2. Inclusion criteria for enrolment 29](#__RefHeading___Toc222918717)

[8.3. Exclusion criteria for enrolment 30](#__RefHeading___Toc222918718)

[8.4. Elimination criteria during the study 30](#__RefHeading___Toc222918719)

[8.5. Contraindications to subsequent vaccination 31](#__RefHeading___Toc222918720)

[8.5.1. Indications for deferral of vaccination 31](#__RefHeading___Toc222918721)

[8.5.2. Absolute contraindications to further vaccination 31](#__RefHeading___Toc222918722)

[8.6. Subject completion and drop out 31](#__RefHeading___Toc222918723)

[8.6.1. Definition of a Drop-out 31](#__RefHeading___Toc222918724)

[8.6.2. Procedures for Handling Drop-outs 32](#__RefHeading___Toc222918725)

[8.6.3. Reasons for Drop-outs 32](#__RefHeading___Toc222918726)

[9. Investigational Products and Administration 32](#__RefHeading___Toc222918727)

[9.1. The MSP 3 LSP study vaccine 32](#__RefHeading___Toc222918728)

[9.2. The Hepatitis B Control Vaccine 33](#__RefHeading___Toc222918729)

[10. Vaccine administration 34](#__RefHeading___Toc222918730)

[11. Prior and Concomitant Therapy 34](#__RefHeading___Toc222918731)

[12. Management of Vaccines 34](#__RefHeading___Toc222918732)

[12.1.1. Labelling and Packaging 34](#__RefHeading___Toc222918733)

[12.1.2. Storage and Shipment Conditions 35](#__RefHeading___Toc222918734)

[12.1.3. Accountability 36](#__RefHeading___Toc222918735)

[12.1.4. Return of Unused Products 36](#__RefHeading___Toc222918736)

[13. Adverse events management and reporting 37](#__RefHeading___Toc222918737)

[13.1. Definitions 37](#__RefHeading___Toc222918738)

[13.1.1. Adverse Event (or Adverse Experience) 37](#__RefHeading___Toc222918739)

[13.1.2. Serious Adverse Event 37](#__RefHeading___Toc222918740)

[13.2. Clinical laboratory parameters and other abnormal assessments qualifying as adverse events and serious adverse events 38](#__RefHeading___Toc222918741)

[13.3. Management of Adverse events 39](#__RefHeading___Toc222918742)

[13.4. Safety Data Collection and Reporting 39](#__RefHeading___Toc222918743)

[13.4.1. Expected Adverse Vaccine Reactions 39](#__RefHeading___Toc222918744)

[13.4.2. Safety Data Collection 39](#__RefHeading___Toc222918745)

[13.4.2.1. Collection and follow-up of adverse events 39](#__RefHeading___Toc222918746)

[13.4.2.2. Assessment of intensity 39](#__RefHeading___Toc222918747)

[13.4.2.3. Assessment of causal relationship 39](#__RefHeading___Toc222918748)

[13.4.2.4. Assessment of Outcome 39](#__RefHeading___Toc222918749)

[13.4.3. Reporting of Serious Adverse Events 39](#__RefHeading___Toc222918750)

[13.5. Regulatory requirements 39](#__RefHeading___Toc222918751)

[13.6. Blood sampling 39](#__RefHeading___Toc222918752)

[13.7. Lost to Follow-up Procedures 39](#__RefHeading___Toc222918753)

[13.8. Specific case: Diagnosis and treatment of Malaria cases 39](#__RefHeading___Toc222918754)

[13.8.1. Diagnosis 39](#__RefHeading___Toc222918755)

[13.8.2. Malaria case management 39](#__RefHeading___Toc222918756)

[14. Primary evaluation criteria 39](#__RefHeading___Toc222918757)

[14.1. Definition of the Criterion 39](#__RefHeading___Toc222918758)

[14.2. Parameters to be measured 39](#__RefHeading___Toc222918759)

[14.2.1. Solicited symptoms 39](#__RefHeading___Toc222918760)

[14.2.1.1. Local reactions 39](#__RefHeading___Toc222918761)

[14.2.1.2. Systemic reactions 39](#__RefHeading___Toc222918762)

[14.2.2. Unsolicited symptoms 39](#__RefHeading___Toc222918763)

[15. Secondary Evaluation Criteria: Immunogenicity 39](#__RefHeading___Toc222918764)

[15.1. Humoral Immune response 39](#__RefHeading___Toc222918765)

[15.1.1. Definition of the Criteria 39](#__RefHeading___Toc222918766)

[15.1.2. Parameters to be measured 39](#__RefHeading___Toc222918767)

[15.1.3. Method and Timing of Measurement 39](#__RefHeading___Toc222918768)

[15.2. Cellular Immune response 39](#__RefHeading___Toc222918769)

[15.2.1. Definition of the Criteria 39](#__RefHeading___Toc222918770)

[15.2.2. Method and timing 39](#__RefHeading___Toc222918771)

[15.2.3. Parameters to be measured 39](#__RefHeading___Toc222918772)

[15.3. Exploratory assays: Functionality of immune response 39](#__RefHeading___Toc222918773)

[15.3.1. Native protein recognition on Merozoite 39](#__RefHeading___Toc222918774)

[15.3.2. Antibody Dependant Cytophilic Inhibition 39](#__RefHeading___Toc222918775)

[16. Statistical Methods and Data Analysis 39](#__RefHeading___Toc222918776)

[16.1. Principal Objective 39](#__RefHeading___Toc222918777)

[16.2. Sample size considerations 39](#__RefHeading___Toc222918778)

[16.3. Data Set to be analysed 39](#__RefHeading___Toc222918779)

[16.3.1. Definition of Population 39](#__RefHeading___Toc222918780)

[16.3.1.1. Total Vaccinated Cohort 39](#__RefHeading___Toc222918781)

[16.3.1.2. According to protocol (ATP) cohort for analysis of safety 39](#__RefHeading___Toc222918782)

[16.3.1.3. According to protocol (ATP) cohort for analysis of immunogenicity 39](#__RefHeading___Toc222918783)

[16.4. Statistical Methods 39](#__RefHeading___Toc222918784)

[16.5. Data Management 39](#__RefHeading___Toc222918785)

[17. Ethical Considerations 39](#__RefHeading___Toc222918786)

[17.1. Informed Consent 39](#__RefHeading___Toc222918787)

[17.2. Risks and benefits 39](#__RefHeading___Toc222918788)

[17.3. Compensation 39](#__RefHeading___Toc222918789)

[17.4. Ethical review and approval 39](#__RefHeading___Toc222918790)

[18. Quality assurance and quality control 39](#__RefHeading___Toc222918791)

[18.1. Access to documents 39](#__RefHeading___Toc222918792)

[18.2. Study personnel 39](#__RefHeading___Toc222918793)

[18.3. Changes to Protocol 39](#__RefHeading___Toc222918794)

[18.4. Investigational procedures 39](#__RefHeading___Toc222918795)

[18.5. Monitoring 39](#__RefHeading___Toc222918796)

[18.5.1. Pre-trial and study initiation visits 39](#__RefHeading___Toc222918797)

[18.5.2. Routine monitoring visits 39](#__RefHeading___Toc222918798)

[18.5.3. Study close out visit 39](#__RefHeading___Toc222918799)

[18.6. Audits 39](#__RefHeading___Toc222918800)

[19. After the trial 39](#__RefHeading___Toc222918801)

[20. Publication and results dissemination 39](#__RefHeading___Toc222918802)

[21. Appendices 39](#__RefHeading___Toc222918803)

**PROTOCOL SYNOPSIS**

| **Sponsor legal name** | African Malaria Network Trust |
| --- | --- |
| **Finished product** | MSP 3 LSP malaria vaccine |
| **Active ingredient** | MSP 3 [181-276) LSP |
| **Trial Title:** | A double blind, randomized, controlled, dose escalation phase Ib field trial in 12 to 24 month old children in Burkina Faso to evaluate the safety and immunogenicity of the *Plasmodium falciparum* Merozoite Surface Protein-3 Long synthetic peptide (MSP 3) adjuvanted in aluminium hydroxide versus hepatitis B |
| **Trial Identifier:** | **MSP 3_BF_0302** |
| **Clinical phase** | Phase 1b |
| **Principal**  **Investigator** | Dr. Sodiomon Bienvenu Sirima, BA, MD, PhD |
| **Investigators** | Alfred Tiono MD  Amadou T. Konate, MD, PhD  Alphonse Ouedraogo, MD  Adama Gansané, Pharm D, MSc  Issiaka Soulama Pham D, MSc  Edith Bougouma Pharm D |
| **Biological**  **Evaluators**  **Biological supervisor** | Issa Nebie, MSc, PhD  Amidou Diarra MSc. |
| **Trial Centre(s)** | **Centre National de Recherche et de Formation sur le Paludisme**  01 BP 2208 Ouagadougou 01  BURKINA FASO  Tel : (226) 50 32 46 95/96 Fax : (226) 50 30 52 20 |
| **Planned Trial**  **Period** | March 2007 to February 2008 |
| **Primary Objective** | **To assess the safety and reactogenicity** of 3 doses of 15 µg and 30 µg MSP3 adjuvanted in aluminium hydroxide given at D0, D28 and D56 in healthy children aged 1-2 years old in Burkina Faso on the following :   1. Solicited adverse events    - immediate reactogenicity (within 1 hour, with emphasis on allergic reactions),    - local and systemic reactogenicity during 7 days following the vaccine injection (day of vaccination and 6 consecutives following days) 2. Unsolicited adverse events occurring within 28 days following each vaccination 3. Serious adverse events (SAE) whenever it occurs during the trial period (from first visit to last visit). |
|  |  |
| **Secondary**  **Objective** | **To assess the humoral immune response** to the vaccine antigen by measuring the increase in the level of antibodies **through measuring:**  IgG, IgM and IgA to MSP3-LSP and peptides a, b, c and d of MSP3  Isotypes IgG1, IgG2, IgG3, IgG4, (MSP3-LSP peptide)  Using ELISA, before and four weeks after each vaccination, as well as 4 months after the last injection and one year after the first injection (D0, D28, D56, D84, D168 and D365)  **To assess the cellular immune response to the vaccine antigens by measuring:**  The number of cells producing IFNγ by Elispot to MSP3-LSP and peptides a,b,c and of MSP3 before and four weeks after the second and the third injection as well as four months after the last injection (D0, D56, D84 and 168) |
| **Exploratory Objective(s)** | To evaluate IgG ability to recognise the native protein on Merozoite by using Western Blot (WB) method before the first injection and four  Weeks, four month and one year after the last injection (D0, D84, D168 and D365)  To evaluate functionality of IgG by using the ADCI technique on D0, D84 D168 and D365 (only if isotype study shows the production of MSP3 specific cytophilic Abs in > 30% of volunteers) |
| **Trial Design** | Randomised, controlled, single trial centre |
| **Planned Sample**  **Size** | The total number of participants will be 45 allocated in two groups as following:   - 22 participants in the full dose (the 30µg of MSP3) or control vaccine group (group 1) - 23 participants in the half dose (15µg of MSP3) or control vaccine group (group 2) |
| **Inclusion Criteria** | 1. Children aged 1-2 years old 2. Healthy by medical history and physical examination 3. Signed Informed Consent by guardian/parent 4. Resident in the study area village during the whole trial period |
| **Non-Inclusion**  **Criteria** | 1. Symptoms, physical signs of disease that could interfere with the interpretation of the trial results or compromising the health of the subjects 2. Immunosuppressive therapy (steroids, immune modulators or immune suppressors) within 3 months prior recruitment. (for corticosteroids, this will mean prednisone, or equivalent, ≥ 0.5 mg/kg/day. Inhaled and topical steroids are allowed.) 3. Cannot be followed for any social, psychological or geographical reasons. 4. Use of any investigational drug or vaccine other than the study vaccine within 30 days preceding the first dose of study vaccine, or planned use up to 30 days after the third dose. 5. Suspected or known hypersensitivity to any of the vaccine components or to previous vaccine. 6. Laboratory abnormalities on screened blood samples out of range, more specifically refer to table 2. 7. Planned administration of a vaccine not foreseen by the study protocol within 30 days before the first dose of vaccine. An exception, is the receipt of an EPI or licensed vaccine (measles, oral polio, Hib, meningococcal and combined diphtheria/pertussis/tetanus vaccines) which may be given 14 days or more before or after vaccination 8. Evidence of chronic or active hepatitis B infection 9. Presence of chronic illness that, in the judgement of the investigator, would interfere with the study outcomes or pose a threat to the participant’s health. 10. Administration of immunoglobulin and/or any blood products within the three months preceding the first dose of study vaccine or planned administration during the study period 11. History of surgical splenectomy. 12. Moderate or severe malnutrition at screening defined as weight for age Z-score less than ‑2 |
| **Investigational Product** | MSP 3 [181-276] LSP |
| ***Form*** | Adjuvanted with Alhydrogel in lyophilised form |
| ***Dose*** | 30µg or 15µg |
| ***Route*** | Subcutaneous, alternate deltoid region |
| **Control Product** | Hepatitis B |
| **Vaccination**  **Schedule** | Primary immunisation : day 0, 28; and 56 |
| **Follow-up duration** | One year after the first vaccination |
| **Blood sampling Schedule** | **D-28: (**screening)  **D0, D28 and D56**: (immediately before each vaccination)  **D84:** (28 days after third vaccination)  **D168:** (4 months after last vaccination)  **D 365:** (1 year after first vaccination) |
| **Primary**  **Evaluation Criteria** | The primary endpoints are measures of the safety and reactogenicity of the vaccine as determined by:   1. Occurrence of solicited symptoms during a 7 day follow-up period after each vaccination (day of vaccination and 6 consecutives days) 2. • Occurrence of unsolicited symptoms during a 28 day follow-up period after each vaccination 3. • Occurrence of serious adverse events throughout the study period |
| **Secondary Evaluation Criteria** | 1. **Humoral response to vaccine antigens:**   Post vaccination levels of IgG, IgA and IgM, expressed in arbitrary units (ng/ml), relative to the pre-vaccination level (D0): D28/D0, D56/D0, D84/D0, D168/D0, and D365/D0.   1. **Cellular immune response to vaccine antigens:**   The number of cells producing IFNγ by Elispot expressed in number of spots /10^6^ cells |
| **Exploratory Evaluation Criteria** | **To evaluate IgG ability to recognise the native protein on Merozoite by using WB**   - **Parasite inhibition by ADCI (SGI %) on D0, D84, D168 and D365. (only if isotype study shows the production of MSP3 specific cytophilic Abs in > 30% of volunteers)** |
| **Statistical Methods** | The analysis shall be descriptive  The final analysis is planned 1 month post Dose 3 to evaluate whether the safety GO/No GO criteria are met to proceed to a phase 2b in. An annex safety analysis will be performed at the end of the study, 9 months post Dose 3. |

| Study phases | Screening | Vaccination Phase (Double blind) | | | | | | | | | | Follow up (Single blind) | | |
| --- | --- | --- | --- | --- | --- | --- | --- | --- | --- | --- | --- | --- | --- | --- |
| Clinic Visit | **V1** | **V2** |  | **V3** | **V4** |  | **V5** | **V6** |  | **V7** | **V8** | **V9** |  | **V10** |
| **Home visits^1^** |  |  | **HV1-5** |  |  | **HV6-10** |  |  | **HV11-15** |  |  |  | **HV16-21** |  |
| **Trial Timelines (Days,)** | **D-28** | **D0** | **D1-D5** | **D6** | **D28** | D29-D35 | **D36** | **D56** | **D57-D62** | **D63** | **D84** | **D168** | **D196-D337** | **D365** |
| **Time Windows (Days)** |  | ± 4 D |  |  | ± 4 D |  |  | ± 4 D |  |  | ± 4 D | ± 7 D | ± 7 D | ± 14 D |
| **Study procedures** |  |  |  |  |  |  |  |  |  |  |  |  |  |  |
| Screening consent | ⌧ |  |  |  |  |  |  |  |  |  |  |  |  |  |
| Study Consent |  | ⌧ |  |  |  |  |  |  |  |  |  |  |  |  |
| Check of inclusion /non Inclusion Criteria | ⌧ | ⌧ |  |  |  |  |  |  |  |  |  |  |  |  |
| Medical History | ⌧ | ⌧ |  |  |  |  |  |  |  |  |  |  |  |  |
| Worm infestation treatent | ⌧ |  |  |  |  |  |  |  |  |  |  |  |  |  |
| Physical Examination | ⌧ | ⌧ |  | ⌧ | ⌧ |  | ⌧ | ⌧ |  | ⌧ | ⌧ | ⌧ |  | ⌧ |
| Check of contra-Indications to vaccination |  | ⌧ |  |  | ⌧ |  |  | ⌧ |  |  |  |  |  |  |
| Randomisation |  | ⌧ |  |  |  |  |  |  |  |  |  |  |  |  |
| Vaccine administration |  | **⌧** |  |  | **⌧** |  |  | **⌧** |  |  |  |  |  |  |
| Check of elimination criteria |  | ⌧ |  | ⌧ | ⌧ |  | ⌧ | ⌧ |  | ⌧ | ⌧ | ⌧ |  | ⌧ |
| Recording of medication |  | ⌧ | ⌧ | ⌧ | ⌧ | ⌧ | ⌧ | ⌧ | ⌧ | ⌧ | ⌧ |  |  |  |
| **Safety and reactogenicity data collection** |  |  |  |  |  |  |  |  |  |  |  |  |  |  |
| Immediate post-vaccine surveillance (60’) |  | ⌧ |  |  | ⌧ |  |  | ⌧ |  |  |  |  |  |  |
| Recording of solicited Local & Systemic Events/Reactions |  | ⌧ | ⌧ | ⌧ | ⌧ | ⌧ | ⌧ | ⌧ | ⌧ | ⌧ |  |  |  |  |
| Recording of unsolicited adverse events |  | ⌧ | ⌧ | ⌧ | ⌧ | ⌧ | ⌧ | ⌧ | ⌧ | ⌧ | ⌧ |  |  |  |
| Recording of Serious Adverse Events |  | ⌧ | ⌧ | ⌧ | ⌧ | ⌧ | ⌧ | ⌧ | ⌧ | ⌧ | ⌧ | ⌧ | ⌧ | ⌧ |
| **Laboratory analysis** |  |  |  |  |  |  |  |  |  |  |  |  |  |  |
| Complete blood count | ⌧ | ⌧ |  | ⌧ | ⌧ |  | ⌧ | ⌧ |  | ⌧ | ⌧ |  |  |  |
| Creatinine, ALT/AST, Biluribin | ⌧ | ⌧ |  | ⌧ | ⌧ |  | ⌧ | ⌧ |  | ⌧ | ⌧ |  |  |  |
| Humoral response measurement |  | ⌧ |  |  | ⌧ |  |  | ⌧ |  |  | ⌧ | ⌧ |  | ⌧ |
| Cell mediated immunity measurement |  | ⌧ |  |  |  |  |  | ⌧ |  |  | ⌧ |  |  | ⌧ |
| Functional assays (ADCI) |  | ⌧ |  |  |  |  |  |  |  |  | ⌧ | ⌧ |  | ⌧ |
| Anti-HBs antibodies |  |  |  |  |  |  |  |  |  |  |  | ⌧ |  |  |
| Western blot |  | ⌧ |  |  |  |  |  |  |  |  | ⌧ | ⌧ |  | ⌧ |
| **Analysis and reporting** |  |  |  |  |  |  |  |  |  |  |  |  |  |  |
| Final Analysis |  |  |  |  |  |  |  |  |  |  | ⌧ |  |  |  |
| Annex safety Analysis |  |  |  |  |  |  |  |  |  |  |  |  |  | ⌧ |

**^1^**  from the first home (HV1) to HV15 the visit will be daily. The visits wil be monthly from HV16 to HV21

| **List of Abbreviations** |
| --- |

95% CI 95% Confidence Interval

AIDS Acquired Immunodeficiency Syndrome

AMANET African Malaria Network Trust

ADCI  Antibody Dependent Cellular Inhibition of parasite growth assay

CRF  Case Report Form

CNRFP Centre National de Recherche et de Formation pour le Paludisme

CTA Centre des Traitements Ambulatoires

DSMB Data and Safety Monitoring Board

EC  Ethics Commitee

ELISA Enzyme Linked Immuno Sorbent Assay

EMVI European Malaria Vaccine Initiative

GCP Good Clinical Practice

GMC Geometric Mean Concentration

GPT Glutamic-Pyruvic Transaminase

HBV  Hepatitis B Virus

HCV  Hepatitis C Virus

HIV Human Immunodeficiency Virus

IgG Immunoglobuline G

INFγ  Interferon γ

MCHC Mean Corpuscular Hemoglobin Concentration

MCH Mean Corpuscular Hemoglobin

MCV Mean Corpuscular Volume

MSP3 Merozoite surface Protein 3

RBC  Red Blood Cells

RESA Ring Erythrocyte Surface Antigen

SAE  Serious Adverse Events

SOP  Standardised Operating Procedure

SGOT Serum Glutamic Oxaloacetic Transaminase

WB Western Blot

WBC White Blood Cells

WHO World Health Organization

# Background Information

## Introduction

Among the four species of Plasmodium that cause human malaria, *P. falciparum* is responsible for most disease and death from malaria. Its life cycle is complex. Disease occurs as a result of the asexual blood stage when parasites invade and grow inside red blood cells. *P. falciparum* virulence is partially explained by its ability to use various receptor pathways to invade red blood cells of all ages. Red blood cells infected with *P. falciparum* bind to endothelium or placenta allowing the parasite to avoid spleen-dependent killing mechanisms but contributing much to pathogenesis^1^.

Anopheline mosquitoes inject sporozoites into the subcutaneous tissue and less frequently directly into the blood stream. The sporozoites then travel to the liver, where they invade hepatocytes. Under two weeks later, each infected hepatocytes releases 20,000-40,000 merozoites into the bloodstream. Despite the destruction of liver cells, no disease results from the infection during parasite development within hepatocytes. Only the blood stages of the infection produce clinical symptoms and malaria disease.

Red blood cells are invaded through a determined sequence. *P. falciparum* must engage receptors on red cells for binding^2^ and undergo apical reorientation, junction formation, and signaling^3,4^. The parasite then induces a vacuole derived from the red cell plasma membrane and enters the vacuole by a moving junction. Within this parasitophorous vacuole *P. falciparum* develops over 48 hours producing around 17-32 merozoites, each able to invade other red cells. Particular to *P. falciparum* is its ability to modify the surface of the red blood cell in a way that the infected cells can adhere to the vascular endothelium and other tissues, where they may cause disease. Parasite sequestration in various organs (brain, heart, liver, kidney and placenta) contributes to the pathogenesis of malarial disease.

Following a number of intra-erythrocytic cycles, a small proportion of asexual parasites convert to gametocytes that are critical for the transmission of the infection to others through female Anopheline mosquitoes. Gametocytes cause no disease and there is no known induced natural immune response to this intracellular sexual form of the parasite.

The strongest natural defence against this parasite is observed in exposed individuals from hyper-endemic malaria areas. Humans acquire a protective immunity, non-sterilizing and not dependant on the parasite strain. I*n vivo* transfer of immune African antibodies to non-protected, *P. falciparum* ridden individuals considerably reduces the level of parasites and protects these subjects^5^. Defence mechanisms studies show that these protective antibodies work in a “roundabout” way, through an antibody-dependant cellular inhibition mechanism (ADCI), which causes monocytes to offload parasitostatic substances. This mechanism, has only been observed with IgG1 and IgG3 cytophilic immunoglobulin isotypes, and is now considered a major stage in the acquisition of a protection against *P. falciparum* clinical malaria^6, 7, and 8^. Unfortunately, this natural immunity to malaria takes a long time, with repeated attacks to develop; and this makes the children born in endemic areas especially vulnerable to attacks of malaria.

With the apparent failure of current control measures as evidenced by the increasing burden of malaria, there is an urgent need to develop more tools for fighting the scourge of malaria. An effective vaccine against malaria would hold such a promise.

The vaccine strategy developed in this protocol is based on the observation of human *P. falciparum* interactions and consists of three steps: i) limited, ethically acceptable, clinical experiments in order to identify the immune mechanisms underlying *in vivo* clinical events, ii) *in vitro* experiments based on these mechanisms in order to identify target antigens, iii) *in vivo* studies in order to assess the role of these antigens in the protection acquired in humans.

The antibody-dependant cellular inhibition method (ADCI) was used as a mean of selecting molecules able to induce protective immunity to malaria. An antibody specific mechanism which promotes parasite killing mediated by monocytes was then identified in sera of clinically protected individuals. These antibodies are specific to the surface protein of the Merozoite (MSP3), a protein of 48kDa molecular weight^9^. Purified immunoglobulins from the protected individuals are confirmed to be efficient in ADCI and the ones that are specifically directed against MSP3 are mainly cytophilic. On the contrary, in the unprotected individuals whose antibodies are not ADCI effective, anti-MSP3 antibodies are essentially non-cytophilic^10, 11^.

The MSP3 region that induces ADCI effective antibodies was highlighted and its sequence defined (MSP3b). It contains an epitope that is not defined by a repetitive structure, and so seems to be non-polymorphic and well conserved. Antibodies induced in mice against this epitope, as well as human antibodies immuno-purified on this peptide, elicit a strong inhibition of *P. falciparum* growth in ADCI assay, whilst control antibodies, directed to peptides from other molecules, do not^10, 11, 12^. The correlation between isotypes of antibodies produced against the 48 kDa epitopes, clinical protection, and ability of specific anti-MSP3 antibodies to block the parasite schizogony in the ADCI assay suggested that this molecule is involved in eliciting protective mechanisms. Moreover, in contrast with other vaccine candidates, the target B and T-cell epitopes were found to be fully conserved in 67 *P.falciparum* isolates^13^.

## Epidemiology

Malaria is a leading cause of morbidity and mortality and an important contributor of poverty in Africa where it accounts for more than 1 million deaths and an economic cost equivalent to USD 12 billion each year. It has been described as Africa’s public health enemy number one and it is estimated that 90% of global malaria cases and 80% of malaria deaths occur in sub-Saharan Africa. Children under five years of age and women in their first or second pregnancy are the main sufferers. Other vulnerable groups include refugees and short-term visitors from non-endemic areas. The visitors include tourists and investors who are essential for Africa’s economic revival.

In terms of labour charge and drop of economic activity, the cost of malaria is enormous. Worldwide, malaria victims are reported to occupy 3 out of 10 hospital beds; in Africa, where transmission which reaches a peak at harvest time, and affects children under 5 years of age, a “simple” malarial attack amounts to 10 days of work^14^. Thus, malaria is both the poor man’s disease and a cause for poverty. The economic growth of “high transmission countries” has always been inferior to the growth of non-malarial countries.

In Burkina Faso, malaria is endemic. Transmission remains constant, with a peak during the rainy season (June to October), *P.falciparum* is responsible for more than 90% of all clinical malaria cases. The major vectors are *Anopheles gambiae s.s*., *An. arabiensis et An. Funestus*. Children under 5 and pregnant women are the populations at highest risk.

During the five months when transmission reaches a peak, these age-brackets suffer multiple malaria episodes, with an annual malaria death rate reaching 15,000^15^. In the country’s hospitals, malaria is reportedly responsible for 30.7% of all hospitalisations with a mortality rate of 23%^15^.

Balonghin, the selected study area, is a village about 50km out of Ouagadougou. The CNRFP have established a Vaccinology centre within this village. The study participants will come from this village and surrounding ones covered within the CNRFP characterised site with a total population of 100,781 inhabitants.

Of these 18.75 percent are children within the targeted age group for this study. The inhabitants mainly belong to the Mossi (90%) and Fulani (5%) ethnic groups. The adult illiteracy rate is high reaching more than 71%. Populations regard malaria as a major Public Health problem, but the knowledge about this disease and above all, its prevention and the way it should be cured remains inadequate. Traditional medicine still plays an important part in the cure of all diseases as more than 65% of the families use it in the first place.

Unfortunately, control measures currently in use are failing owing to poor healthcare delivery systems, emergence of parasite resistance to cheaper and effective drugs as well as limited effectiveness of vector control due to insecticide resistance and environmental concerns.

Failure in the current control measures call for an accelerated development of malaria vaccines as a potential additional tool in the arsenal against malaria. Reasons for lack of effective interventions for malaria have been eloquently addressed in various publications, but prominent among these is the lack of financial returns to investments on research in the development of new anti-malarial drugs and vaccines.

## Name and description of investigational product

MSP3, a candidate malaria vaccine, is a long synthetic peptide that uses a 181-276 amino acid sequence. It is available in a multi-dose vial presented lyophilised, and looks like amorphous white powder. The vaccine is produced by SYNPROSIS in France.

## Summary of findings from non-clinical studies

***In vivo* passive transfer** experiments in *P. falciparum* infected SCID mice: P. Druilhe *et al*. have recently described *P. falciparum* growth in immuno-deficient mice grafted with human erythrocytes. They report the potential of this new mouse model for vaccine development against *P.falciparum* malaria determined by studying the effect of antibodies in passive transfer experiments, the effect of which is well established in humans^16^. Results show that African adult immunoglobulin strongly reduces *P. falciparum* parasitaemia in a similar fashion to that reported in humans, provided the mice are reconstituted with human monocytes (HuMN). In contrast neither immunoglobulin nor HuMN alone had any direct effect upon the circulating parasitaemia in mice when added separately.

These experiments were extended to assess the *in vivo* effect of epitope-specific antibodies. Human antibodies were affinity-purified on peptides derived either from the Ring Erythrocyte Surface Antigen (RESA) or the Merozoite Surface Antigen-3 (MSP3), and their specificity and titers determined. Results show that the inoculation of low concentrations of anti-MSP3b, but not anti-RESA antibodies, together with HuMN suppress *P. falciparum* parasitaemia in mice in a consistent manner. The rate at which parasitaemia decreased was as fast or faster than that induced by total African IgG, and as fast as that induced by chloroquine^17^. That such a profound biological effect can be obtained using only a minor subset of the total anti-malarial antibodies, i.e. those affinity purified on a single peptide from one of the numerous *P. falciparum* proteins, is striking. However, this result is in keeping with immuno-epidemiological observations, which show a 90% positive predictive value for the level of clinical protection associated with IgG3 antibodies directed to the same MSP3b peptide. The strong effect in mice of small amounts of anti-MSP3 Abs is in agreement with the relatively low levels of the same Abs in populations with clinical protection living in holo-endemic areas. There is therefore a convergence of data obtained by three different means, i.e. under *in vitro* conditions (ADCI), *in vivo* conditions in mice (in Scid), or in humans (by immuno-epidemiological methods). Results demonstrate the value of the model, in which antibodies with distinct specificities can be evaluated sequentially in the same animal, and they also reinforce the potential of MSP3 for vaccine development.

**A recombinant human antibody** was obtained by preparing RNAm from lymphocytes from immune African subjects; cloning cDNA in a filamentous phage capable of producing the Fab’2 immunoglobulin fragments on its surface. Abs specific to MSP3 were isolated by the DG210 antigen through the panning of phages on coated patches. Then, build-ups integrating the heavy-chain genes, namely IgG1 or IgG3, were prepared. Antibodies were produced from CHO cells and then purified. With a concentration of 3mg/l, Ab RAM1 IgG1 produces in vitro an ADCI effect in presence of human monocytes whose activity is equal or superior to that obtained with the African IgG pool. Injected in *P.falciparum* infected SCID mice, it produces complete clearance of parasitaemia within four days whereas an Ac combining an anti-rhesus control has no effect.

**Louis Miller’s team at NIH immunised three series of 7 Aotus**, using either Ag control, or MSP1, or MSP3 with Freund’s adjuvant. Results show partial protection, with maximum parasitaemia of 5%, in 5 out of 7 Aotus receiving MSP1 Stronger protection (maximum parasitaemia 0.9%) was observed in 6 out 7 MSP3 vaccinated monkeys. In MSP3 immunised animals there was a correlation between Ab anti-MSP titres, after immunisation and before challenge, and the observed protection. In MSP1 immunised animals, a) there was no correlation between Ab anti-MSP1 titres and protection, b) a correlation was observed in MSP1 vaccinated animals between challenge driven Ab anti-MSP3s and protection. These results agree with the protection induced in *saimiri* monkeys by MSP3 adjuvanted with Montanide or ASO2^18^.

**The sequence analysis of the MSP3 C-terminal area** was piloted by Australian teams on more than 20 isolates and in Denmark, by M. Theisen on more than 70 isolates. The results show an absolute conservation of the protein sequence and more surprisingly of the nucleotide sequence (no “synonym” codon, that is to say a distinct codon coding for the same amino acid.)

**Studies on MSP3 Immunogenicity** were performed using the MSP3b peptide, three multiple branches peptides (octopus), recombinant DG 210, recombinants covering the C-Terminal expressed in the PGEX vector, the pTCr his vector, the yeast, the Long Synthetic Peptide with a series of adjuvant representative of several existing class: Complete and incomplete Freund adjuvants, alum, ISA 51 and ISA 720 montanide, titermax, Ribi adjuvant, one of Aventis’s experimental adjuvants, one from OM-pharma, GSK’s SBAS2, and the priming combination by MSP3-PPD BCG-boosting. In mice, except for alum, all the formulations proved immunogenic to different degrees, some of them were tested on Saimiri and proved to be efficient^5^. Nevertheless, there still wasn’t a parallel in ELISA and in titres on native proteins.

This body of convergent results makes MSP3 a promising vaccine candidate.

## Clinical experience with MSP 3 LSP

**A first phase 1a trial** was conducted in Switzerland, on healthy adults, who had never been exposed to malaria. The MSP3-LSP vaccine was administered to 35 volunteers, in 3 subcutaneous injections at 0, 1 and 4 months. Four different dosages: 10µg, 20µg, 30µg or 100µg and two adjuvants, Montanide 720 and Aluminium hydroxide were evaluated. The following table shows the distribution of subjects by dose and by adjuvant:

| **Groups** | **Dosage** | **Adjuvant** | **Number of subjects** |
| --- | --- | --- | --- |
| M10-10-10 | 10 µg LSP | Montanide 720 | 6 |
| M30-30-10 | 30 µg LSP | Montanide 720 | 6 |
| M100-10-10 | 100 µg LSP | Montanide 720 | 5 |
| M20-20-20 | 20 µg LSP | Montanide 720 | 6 |
| A30-30-30 | 30 µg LSP | Alum | 6 |
| A100-10-10 | 100 µg LSP | Alum | 6 |

Immediate local and systemic tolerance was monitored for the hour following the injection and again two days after vaccination. Humoral and cellular immune responses and the functionality of the immune response were assessed one month after each injection and five and nine months after the booster dose.

The vaccine showed a very good systemic tolerance, whatever the adjuvant or dose, with no systemic reactions observed during the study. In contrast, local tolerance was clearly linked to dose, adjuvant and injection number. Because of large local reactions, with 10cm indurations, the second scheduled 100µg injections were replaced by 10µg injections for both adjuvants. After the second 30µg MSP3, Montanide adjuvated dose, the increasing local reactions observed led the investigator to replace the third dose with a 10µg dose. With a 30µg MSP3 vaccine adjuvanted with Aluminium hydroxide, the growing number of local reactions only emerged after the third dose had been delivered. (See following figures).


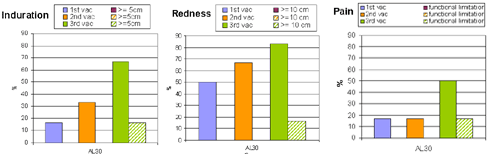


The immune response induced by MSP3’s different formulations in humans, was higher than the response observed in pre-clinical studies. Interestingly, the adjuvant effect of aluminium hydroxide in humans was greater in humans than in monkeys or mice, and it was comparable to the adjuvant effect of Montanide 720. The immune response was maintained nine months after the booster dose was injected. The functionality of the immune response, as measured by the ADCI assay, showed that antibodies induced by vaccination could lead to an inhibition of *P. falciparum.*

To conclude, at a dose of 30µg, the MSP3 vaccine was well-tolerated by healthy adults not exposed to malaria, and led to the production of antibodies capable, in vitro, of neutralizing the *P. falciparum* parasite^19^.

**The second study was a phase Ib trial** conducted in Burkina Faso among semi-immune adults. A total of 30 participants enrolled ad all of them received three vaccine doses in the same schedule of 0, 1 and 4 months. All participants completed the study *per protocol*.

No immediate local reactions of any kind were recorded after any of the three vaccinations. During the 14 days follow-up period after each vaccination no local reactions that prevented normal daily activity were recorded. The most frequently reported local symptom in both vaccine groups was mild swelling (size less than 3 cm) at the injection site lasting up to 6 days after the vaccination. Only one participant in the tetanus group reported a mild induration lasting 16 days which resolved without sequellae. Local adverse reactions/events were recorded with similar frequency in the two vaccine groups.

Only one participant in the MSP3-LSP group experienced an immediate systemic reaction, an episode of mild tachycardia, possibly related to the vaccination, which lasted for 2 hours. No action was taken and this was not considered to be a serious adverse event.

Two individuals in the MSP3-LSP vaccine group had a malaria episode compared with one individual in the tetanus group. The most frequent syndrome was bronchitis (7 cases in the MSP3-LSP group and 4 cases in the tetanus group). No individuals experienced diffuse erythema, urticaria, Quincke’s oedema, asthma, malaise, drowsiness, experienced pallor, sweating, tachycardia (except the immediate reaction described above), loss of appetite, flu-like syndrome. All symptoms resolved without sequellae.

Eosinophilia was observed in all participants at some point during follow-up except one, with similar frequency in the two vaccine groups. Two individuals in the MSP3-LSP group and one in the tetanus group experienced hyperglycaemia. In all three individuals the hyperglycaemia was transient and in none was it considered clinically significant. None of the haematological abnormalities were judged to be related to the study vaccines.

|  | **MSP-3 LSP*** | | | **Tetanus*** | | |
| --- | --- | --- | --- | --- | --- | --- |
|  | Dose 1  N=15 | Dose 2  N=15 | Dose 3  N=15 | Dose 1  N=15 | Dose 2  N=15 | Dose 3  N=15 |
| Pain | 6 | 5 | 5 | 7 | 8 | 3 |
| Erythema | 0 | 1 | 1 | 1 | 1 | 0 |
| Induration | 0 | 2 | 3 | 3 | 4 | 3 |
| Swelling | 4 | 8 | 7 | 8 | 6 | 6 |
| Pruritus | 2 | 0 | 1 | 1 | 0 | 3 |
| Local heat | 0 | 0 | 0 | 0 | 0 | 0 |

In summary, both vaccines were well tolerated; no unexpected adverse reactions were reported. No serious adverse events were observed. The reactogenicity profile of the MSP3-LSP vaccine did differ substantially from that of tetanus vaccine in male adults.

No detectable Humoral immune response was induced by the MSP3-LSP vaccine. Lympho-proliferation in response to stimulation with PHA antigen was similar in the two vaccine groups. The response in the tetanus vaccine group to stimulation with tetanus toxoid antigen increased following vaccination, while the response in the MSP3-LSP group was unchanged after vaccination. The response in the MSP3-LSP vaccine group to stimulation with MSP3-LSP antigen increased after vaccination, while that in the tetanus group appeared unchanged

The pattern of IFN gamma responses to stimulation with PHA antigen was very similar in the two groups with a slight decrease between Day 0 and Day 56. IFN gamma responses to stimulation with tetanus toxoid antigen were unchanged post vaccination. IFN gamma responses to stimulation with MSP3-LSP antigen appeared to remain stable in the MSP3-LSP group after vaccination, while in the tetanus group, they showed a similar decline to that observed with PHA.

## Rationale

The phase 1 trial results in young Swiss adults highlight the MSP molecule’s vaccine potential. As the next step in the clinical development of this vaccine, the phase Ib trial in Burkina Faso has further demonstrated that the MSP 3 LSP vaccine is safe even in populations exposed to *P. falciparum.* A desirable property of the vaccine shown through the second study was that the local reactogenicity in *P.falciparum* exposed individuals after the first injection was less than that observed after the second and third doses in Swiss volunteers.

Having met the preset go criteria for the adult studies, the vaccine now requires approaching the ultimate target population in a stepwise manner. A vaccine intended for developing countries, can only be realistically deployed through the Expanded Programme on Immunisation (EPI). In order to fit in that programme, the schedule of administering our vaccine has to be changed so that it could fit in. All the clinical experience with this vaccine so far has been with adults. We have decided to be cautious and not presume its safety in children; therefore, both the 30µg and 15µg of the MSP 3 LSP vaccine will be evaluated. Hepatitis B vaccine has been selected as control vaccine for the following technical and ethical reasons:

- It has recently been adopted and incorporated within the Burkina Faso EPI immunisation programme, and therefore is clearly of public health benefit;
- Can be administered in a schedule acceptable for evaluating our study vaccine;
- The targeted children in this trial would not have received it; therefore it will specifically be on benefit to them.

# Trial Objectives

## Primary Objective

**To assess the safety and reactogenicity** of 3 doses of 15 µg and 30 µg MSP3 LSP adjuvanted in Aluminium hydroxide given at D0, D28 and D56 in healthy children 1-2 years old in Burkina Faso on the following:

1. Immediate reactogenicity (within 1 hour, with emphasis on allergic reactions),
2. Local and systemic reactogenicity during the 7 days following the vaccine administration(day of vaccination and 6 subsequent following days)
3. Unsolicited adverse events occurring within 28 days following each vaccination
4. Serious adverse events (SAE) throughout the study period.

## Secondary Objectives

1. To assess the humoral immune response to the vaccine antigen by measuring the increase in the level of antibodies through measuring:

IgG, Ig M and IgA to MSP3-LSP and to MSP3 peptides (a,b,c and d )

Isotypes IgG1, IgG2, IgG3, IgG4, (MSP3-LSP peptide)

Using ELISA, before and four weeks after each vaccination, as well as 4 and 9 months after the last injection

1. **To assess the cellular immune response** to the vaccine antigens by measuring: the number of cells producing IFNγ/10^6^ cells by Elispot to MSP3-LSP and MSP3 peptides a, b, c and d

Before and four weeks after the second and the third injection

## Tertiary or exploratory objectives

1. To evaluate IgG ability to recognise the native protein on Merozoite by using Western Blot method.
2. To evaluate functionality of IgG by using the ADCI technique.

# Study Design Overview

| **Study Days** | | D0 | | D14 | | D28 | | | D42 | | D56 | | D70 | | D84 | D98 |  |  |  |
| --- | --- | --- | --- | --- | --- | --- | --- | --- | --- | --- | --- | --- | --- | --- | --- | --- | --- | --- | --- |
|  | |  | |  | |  | | |  | |  | |  | |  |  |  |  |  |
|  |  |  |  | | |  |  | | | |  | | | |  |  |  | |  |
|  |  |  |  | | |  |  | | | |  |  | | | Group 1 (15µg) | |  | |  |
|  |  |  | | | |  | | | | |  | | | |  | |  | |  |
|  |  | Vacc 1 | | | | Vacc 2 | | | | | Vacc 3 | | | |  | |  | |  |
|  |  |  | |  | |  | | |  | |  | |  | |  |  |  | |  |
|  |  |  | |  |  | | | |  |  | | |  | | | Group 2 (30µg) | | |  |
|  |  |  | |  | |  | | |  | |  | |  |  | |  |  | |  |
|  |  |  | | Vacc1 | |  | | | Vacc2 | |  | | Vacc3 |  | |  |  | |  |
|  |  |  | | | |  | |  |  | |  | | |  | | |  |  |  |
|  | |  | | | |  | | | | |  | | |  | | |  | |  |
|  | |  | Safety reports post 7 days follow up | | | | | | | | Vacc = Vaccination | | |  | | |  | |  |

|  |  | | |  | |  |  |  |  | |  | |  | |  |  |  |  | |  |
| --- | --- | --- | --- | --- | --- | --- | --- | --- | --- | --- | --- | --- | --- | --- | --- | --- | --- | --- | --- | --- |
|  |  | | |  | |  |  |  |  | |  | |  | |  |  |  |  | |  |
|  |  | | |  | |  |  |  |  |  |  |  |  |  |  |  |  |  | |  |
|  |  | | |  | |  |  | **Vacc 1** |  | | **Vacc 2** | | **Vacc 3** | |  | BS |  | BS | BS |  |
|  |  | | |  | | **Randomization** | | *MSP3 BS* |  | | *MSP3 BS* | | *MSP3 BS* | |  |  |  |  | |  |
|  |  | | |  | |  |  | |  | |  | |  |  |  |  | |  |
|  |  | | |  | |  |  | |  | |  | |  |  |  |  | |  |
| BS |  | | |  | |  |  | |  | |  | |  |  |  |  | |  |
|  |  | | |  | |  |  | |  | |  | |  |  |  |  | |  |
|  |  | | |  | |  |  |  |  | |  | |  | |  |  |  |  | |  |
|  |  | | |  | |  |  |  |  |  |  |  |  |  |  |  |  |  | |  |
|  |  | | |  | |  |  | **Vacc 1** |  | | **Vacc 2** | | **Vacc 3** | |  | BS |  | BS | BS |  |
|  |  | |  | |  | |  | *Hepatitis B BS* |  | | *Hepatitis B BS* | | *Hepatitis B BS* | |  |  |  |  | |  |
|  |  | | | | |  |  |  |  | |  | |  | |  |  |  |  | |  |
|  |  | | |  | |  |  |  |  |  |  |  |  |  |  |  |  |  | |  |
| **Clinic  Visit 1**  Day -28 to -14 | |  | | | |  |  | **Clinic  Visit 2**  Day 0 |  | | **Clinic  Visit 3**  Day 28 | | **Clinic  Visit 4**  Day 56 | | **Clinic  Visit 5** Day 84 | | **Clinic  Visit 6** Day 168 | **Clinic  Visit 7** Day 365 | |  |
|  |  | | | | |  |  |  |  | |  | |  | |  | |  |  | |  |
|  | **SCREENING** | | | | | | | **VACCINATION** | | | | | | | | | **LONG TERM SAFETY FOLLOW UP** | | |  |
|  |  | | | | | | | **DOUBLE-BLIND PHASE** | | | | | | | | | **SINGLE-BLIND PHASE** | | |  |
|  |  | | | | |  |  |  |  | |  | |  | |  | |  |  | |  |
| **BS;** Blood Sample. **Vacc;** Vaccination. | | | | | | | | | | | | | | | | | | | | |
|  | | | | | | | | | | | | | | | | | | | | |

- Double blind (observer blind, participant blind), randomized, controlled, dose escalation, Age de-escalation, phase Ib study
- One study centre,
- Two parallel groups
- Group 1: 23 subjects (15 subjects receiving MSP-3 vaccine 15 µg and 8 subjects receiving Hepatitis B vaccine).
- Group 2: 22 subjects (15 subjects receiving MSP-3 vaccine 30 µg and 7 subjects receiving Hepatitis B vaccine)
- Immunization schedule will be 0, 1, and 2 months for all cohorts and provisionally as following for each group:
  - Study days 0, 28 and 56 for group 1
  - Study days 14, 42, 70 for group 2
- Vaccinations of groups 1 and 2 will be staggered: immunization in group 2 will start 2 weeks after group 1. This interval may be extended if deemed necessary due to SAEs or other safety concerns.
- Randomization will be done for each group at the times of first vaccinations.
- Route of inoculation will be by subcutaneous injection into right or left deltoid (alternately).
- Each child will be observed for at least 60 minutes after vaccination to evaluate and treat any acute adverse events (AEs)
- Study duration will be approximately 13 months per subject.
- Seven (7) day follow-up period for solicited adverse events (day of vaccination plus 6 subsequent days)
- Twenty eight (28) day follow-up period for unsolicited adverse events (Vaccination day plus 27 subsequent days)
- Follow-up of serious adverse events (SAE’s) for 12 months after the first dose of study vaccine (9 months after dose 3)
- At the end of the follow-up period for unsolicited AEs (i.e., one month after the third dose), subjects will be followed by field workers at home at monthly intervals to record SAEs.
- Data collection: conventional Case Report Form (CRF).

# Trial design and methodology

## Description and justification of design

The study is a single centre randomised controlled and blinded study (observer blind). It will be conducted at the CNRFP Vaccinology unit located in Balonghin. Children in the catchments area within the 1-2 years age group, whose parents consent will be screened to randomise 45 eligible participants.

Clinical, biological and immune response data gathered after vaccination with 15µg and 30µg MSP 3 LSP will be compared to:

- The children’s baseline data before vaccinations, and
- The post vaccination data of children in the control group.

Randomisation will ensure that the comparison groups are similar in relevant characteristics at baseline. The concealment of allocation before enrolment will further enforce the randomisation. Individuals who will make the assessment of the study end points will be completely blinded of the vaccine administered. This will ensure that there is no observer bias. Further, reporting or information bias will be minimised, because the recipients will also not be aware of which vaccine they have been administered. This is possible because the selected control vaccine has not been in routine use in this area, and has only now been recommended by the Ministry of Health. Cross over immunisation at the end of the trial will involve only those children who will received the study vaccine; they will be administered the control vaccine in the interest of public health benefits for them.

The schedule of vaccination at 0, 1 and 2 months has been adopted because it is suitable for the target group. The idea is to eventually deploy the vaccine through the expanded programme on immunisation should the vaccine become registered for public use. For the EPI age group, it is not only an efficient delivery mechanism, but they are also the most vulnerable group to malaria.

## Study Site

This study will be conducted by the PDVAP/CNRFP. The team at this centre received a grant specifically to build their capacity for undertaking malaria vaccine trials. They have recently built a Vaccinology unit at their field site in Balonghin (50km south of Ouagadougou), which is specifically designed for vaccine trials. The PDVAP/CNRFP has further established demographic surveillance system within Saponé district (100,000 inhabitants living 79 villages around Balonghin). Through this system, data on vital events and basic malariometric parameters have been collected for a deeper and reliable baseline data necessary for potentials malaria phase 2 or 3 vaccines trials.

At the Vaccinology unit, facilities exist for screening many participants, medical examination, blinded randomisation and treatment allocation. There are observation rooms and resuscitation facilities necessary for post vaccination evaluation and treatment of immediate reactions following immunisation. A laminar flow hood is installed for extemporaneous mixing of vaccine with adjuvant. There is constant radio communication available between the site and Ouagadougou where there is the main tertiary hospital. The team has a fully equipped ambulance and 4X4 motor vehicle transport. The PDVAP/CNRFP team has been working in this area for over five years now, and have established a very good relationship with the community. In short, the team is adequately staffed and equipped to conduct this study.

# Conduct of the trial

## Recruitment procedure

From the up to date database of the population in the study area, a list of all children 1-2 years old will be drawn. Parents of selected child will be visited at home by field workers who will discuss the study with them; an invitation letter will be given for them to come to the field station of PDVAP to receive more information about the study. In informed consent procedure will be instituted which will have several levels of authorisations starting from the civil authorities to the local village leadership and down to small groups and ultimately individual parents/guardians. Those who provide a signed informed consent will be screened for study eligibility. A total of 45 eligible children will enrolled

These will further be invited to provide signed and witnessed informed consent before actual enrolment and administration of the study vaccine.

## Randomisation and treatment allocation

### Randomisation of participants

The study statistician will produce a randomisation list before the trial starts. This list will have the exact treatment and dosage allocated to each unique study ID number. The randomisation list for each group will be independent from the other. Subjects will be allocated sequentially to treatment numbers in the order that they present for vaccination.

### Method of blinding and breaking the study blind

Data pertaining to the trial will be collected in a double blinded (observer blinded) manner. “Double blinded (observer blinded)”in this context means that the vaccine recipient and their parent(s)/guardian(s) as well as those responsible for the evaluation of safety and immunogenicity endpoints will all be unaware the exact treatment, (MSP3 or Hepatitis B)given to the participant. The only study staff aware of the vaccine assignment for Hepatitis B or MSP3 will be those responsible for the storage and preparation of vaccines; these staff will play no other role in the study.

. Sealed envelopes labelled with the study ID number and containing the treatment allocated to each particular ID number will be provided to the investigator. The clinical investigator will only be allowed to open an envelope after ensuring that the child before him has met all eligibility criteria and has been given the study ID number. For each child, eligibility will have to be counter checked and signed by a second person before allocation of study ID number.

All envelopes will be retained to be checked by the clinical monitor.

The local safety monitor who is independent from the study team will also be provided a sealed randomisation list corresponding to each group. In total, 2 sealed randomisation list will be provided. If deemed necessary for reasons such as safety, the Local Safety Monitor in Ouagadougou as well as AMANET Central Safety will unblind the specific enrolled subject without revealing the study blind to the investigators.

As part of the safety monitoring plan, safety reports will be produced at 5 timepoints in the trial. An independent statistician will analyze the data, thereby maintaining the blind of the investigator group.

A formal reporting and analysis plan (RAP) will be developed. Once the study is completed and the database locked. AMANET will be responsible for initiating the execution of the statistical analysis plan in collaboration with the investigator team and breaking the blind.

### Trial Calendar

Screening for potential study participants is planned to start in February 2007. The first participant is planned to be recruited in March 2007. The recruitment period will last for four weeks. Vaccinations would then start in March, and will be staggered starting with Lower dose of MSP3 (15 µg) and then the Higher dose (15 µg) 14 days later. Each participant will be followed-up for a total period of one year from the first injection. The study has 11 scheduled clinic visits and 21 scheduled home visits, but participants will be under strict instructions to report at the unit should they feel unwell at any time. The planned final analysis at 1 month post vaccination 3 will look only at safety data to evaluate the go/no go safety criteria for pursuing the clinical development plan.

### Vaccination and serological schedule

Scheduled clinic visits are planned at day -28, 0, 6, 28, 36, 56, 63, 84, 168 and 365. Three vaccine doses will be administered by subcutaneous injection (day 0, 28 and 56). As shown in the flow chart (see page 9), participants will be monitored for the hour following vaccination, then a field worker will visit them at home on each of the 5 days following the injection. The patient will come back to the clinic at the 6^th^ day following each immunization to be assess by a study clinician. Six serological blood sampling points have been planned on days 0, 28, 56, 84, 168 and 365.

Biological evaluations will also be done on days -28, 0, 6, 28, 36, 56, 63, 84 and 365. Apart from these standard examinations, other tests may be performed if deemed necessary.

### Clinical evaluation and follow up

At screening, parents and guardians to all children in the study area aged 4-6 years and 1-2 years will be targeted for information on study participation. Those who are willing and sign the informed consent will undergo a full clinical evaluation including blood sampling for biological screen. Among those emerging as healthy, 45 children fulfilling the study inclusion criteria and who do not have any exclusion criteria, will be selected randomly and invited to participate in the study. The detailed information on this process is provided in the SOP on enrolment of participants.

On every vaccination visits, children will undergo a full physical examination before receiving the injection. Following each injection, they will be observed for one hour to assess for any adverse events following immunisation before they can return to their home. They will further be visited daily at home to evaluate for solicited local and systemic reactions for five days. The final evaluation (day 6) will be done at the clinic.

### Safety monitoring plan

This trial is overseen by a Data Safety Monitoring Board (DSMB), operating under a charter.

The DSMB will be notified of all SAEs within 24 hours. In addition there are defined points during the trial at which cumulative safety data will be reviewed by the DSMB.

The PI, the LSM, and DSMB are empowered to suspend the trial for any safety concern. To supplement this, the protocol defines criteria for the suspension of vaccination.

### Data Safety Monitoring Board (DSMB)

The DSMB for this study is a four member group of skilled professionals constituted according to AMANET procedures that respect international standards. These experts will monitor the progress of the study with particular interest in the safety of trial participants. Their composition, mandate and terms of reference are provided in the annexed DSMB charter.

Briefly, in this study, the DSMB will be responsible for:

- On going safety review and follow up in a unblinded manner
- Receive and review SAE’s when they occur
- Make recommendations to AMANET on the safety balance between the two comparison groups; and
- Make recommendations regarding continuing, amending or termination of the study for safety reasons.

The DSMB will receive progress reports one week after each injection within the first four months. SAE’s will be notified to the chair of the DSMB within 24 hours of the investigator learning of them.

The DSMB must be informed by AMANET as sponsor of the following safety data on an ‘as received’ basis:

- All SAEs;
- All withdrawals of study subjects by the Principal Investigator or the parent(s)/guardian(s) of a subject due to adverse events.

The DSMB will receive from the sponsor, AMANET:

- New information that may affect adversely the safety of the subjects or the conduct of the study;
- All subsequent protocol amendments, ICF changes or revisions of other documents originally submitted for review;
- All subsequent protocol modifications (for information).

The DSMB is empowered to suspend the enrollment to the trial and/or vaccination on the trial pending review of potential safety issues;

### Local Safety Monitor (LSM)

The overall role of the Local Safety Monitor (LSM), who is an experienced clinician based in Ouagadougou, will be to support the clinical investigators and to act as a link between the investigators and the DSMB.

The LSM’s role will include:

- Acting as the study volunteer’s advocate;
- Promptly communicating relevant safety information to the DSMB;
- Providing advice to the investigators on whether a set of clinical circumstances in a study warrants formal notification to the DSMB;
- Unblinding a subject if deemed necessary to allow for adequate treatment;
- Liaising closely with the chair of the DSMB throughout the course of the trial;
- Suspension of vaccination for a major safety concern pending discussion with the DSMB

### Data Reviewed by the LSM

The LSM must be informed by the investigator on an ‘as received’ basis of:

- All SAEs;
- All withdrawals of study subjects by the Principal Investigator or the parent(s)/ guardian(s) of a subject due to adverse events.

### Safety monitoring reports

In each group after all the subjects have completed the 7 days of follow up, a safety report will be produced for the DSMB. In total, 5 safety reports will be provided to the DSMB. An independent statistician will analyze the data and prepare an unblinded report for the DSMB thereby maintaining the blind of the study staff. If a criterion for the suspension of progression of the study is met, or the DSMB have any safety concerns about the vaccines, they may suspend the progression to the next sequential vaccine dose or to the next dose level. The process outlined in Sections 5.2.11 will be followed.

The reports will contain for each group:

- All SAEs and any relationship to vaccine;
- In each group, for each of the first two doses (i.e. after Dose 1 and Dose 2) of MSP3 and Hepatitis B, all solicited AEs tabulated by severity grading (any and Grade 3 alone) and relationship to vaccine;
- In each group, for each of the first two doses (i.e. after Dose 1 and Dose 2) of MSP3 and Hepatitis B, all unsolicited AEs tabulated by severity grading (any and Grade 3 alone) and relationship to vaccine;
- All laboratory values up to 28 days post Dose 2 presented as number of subjects out of range (above and below normal range) at each sampling time point (post doses 1 and 2 of MSP3 and Hepatitis B) tabulated by toxicity grading scale;
- All withdrawals of study subjects by the Principal Investigator due to adverse events recorded from the children or withdrawals of children by the parent(s)/guardian(s) following doses of MSP3 and Hepatitis B (expressed as percentage of subjects enrolled).

To allow the decision making for the progression to the next cohort, a comprehensive cumulative safety report of all the data collected up to date will be submitted to the DSMB in the same format as described above.

### Process for the suspension of progression to the next sequential vaccine dose or to the dose escalation

Suspension of the next sequential vaccine dose or dose escalation, pending full review of available data by the DSMB will take place if:

- The Principal Investigator suspends vaccination for any of the following SAEs pending review by the DSMB;
- Death or life-threatening SAE which is judged to be related to the study vaccine;
- Anaphylactic shock reaction in an enrolled subject following vaccination.
- The DSMB recommend suspension of all vaccination for any one SAE or pattern of SAEs. The DSMB will communicate their recommendation to the Principal Investigator who will enact it. The DSMB will notify the sponsors of their decision immediately;
- A safety report shows > 5% of subjects vaccinated with MSP3 or Hepatitis B are withdrawn by the investigator for local or systemic reactogenicity in recipients;
- In making their recommendation the DSMB will take into account the full clinical history of each withdrawn child.

### Process if the trial is suspended on the basis of safety reports

If the trial is suspended, the DSMB will review all available information (which will include the experience of all children to have been vaccinated) and make a recommendation to the study sponsor (AMANET) whether to recommence the trial and proceed to the next vaccination (sequential vaccine dose, or to dose escalation or to the next cohort) or to stop the trial permanently.

Although the trial may be suspended by the DSMB, the LSM or the Principal Investigator, it is the responsibility of the sponsor (AMANET) to make the final recommendation whether or not the trial should be stopped permanently.

## Termination of the trial

AMANET as the trial sponsor reserves the right to terminate this study. The trial may be discontinued for the following reasons:

1. If new data about the investigational product resulting from this or any other trials become available that dictates that the product is not safe for human use;
2. When AMANET so advises based on administrative reasons or other advices from the DSMB;
3. When the National Ethics Committee so decides.

If a trial is to be prematurely terminated or suspended, AMANET shall promptly inform the investigators, the Regulatory Authorities and the Ethics Committee of the reason for termination.

Should the principal investigator deem termination inevitable, he may suspend study activities while immediately resolving the issues raised with AMANET.

# Data collection and handling

## Source data

All records bearing participant information which is generated during this study shall constitute primary or source documents. This includes the files opened for the participants as record files, hospital files, study log books, dairy cards, and hospital files in case of hospitalisation. All laboratory result sheets will also be considered as source documents. A list of agreed source documents will be maintained in the investigator file, and these shall be used for verifying entries on CRF by the monitor.

The investigator shall make every effort to ensure that these documents are maintained and safely kept as advised by the sponsor considering relevant regulatory requirements.

## Case Report Forms

The CRF constitute important study documentation and will be written on “no carbon required” triple copy paper (white, yellow and pink). Only investigator team officers dully authorised will be allowed to write on CRF and a copy of authorised signatories will be maintained in the study file.

Some of the information, especially participant personal socio-demographic details may be entered directly onto CRF. However, most of the information will be transcribed from source data which will be retained for monitor verification and audit trail. The Principal Investigator takes total responsibility to ensure that correct information is entered on the CRF and shall sign on each CRF to this effect.

Entries shall be made with a black ball point pen. Any corrections made must remain legible, and should only be a neat crossing line, date and initials of the authorised person making the correction and the new entry besides it.

After monitor verification, the original CRF page (white) will be withdrawn and sent to the data management; the yellow page will be shipped to AMANET and the pink page will remain to be stored with the investigator at site together with other essential study documents.

## Data processing

The principal investigator will review and approve of the CRF pages for data entry. The data base for this study is created in Microsoft Access® software and shall be entered by two separate data entrants. The supervising data manager will do all the data reconciliations and validations leading to the validated file for each participant. The database is designed to keep track of all entries in time and password and will be able to provide a complete audit trail.

Any illegible CRF entries will be sent back to the clinical investigators with accompanying appropriate data query form. The monitor will also routinely check the database against the CRFs, and will ensure that all accumulated data query resolutions are verified as consistent on the database, CRF and source document.

Regular reports will be compiled for presenting to the DSMB for their ongoing review of safety data. This shall be in a blinded manner. The final analysis will be done immediately after day 84 to allow for phase IIb study Go/No go evaluation.

At the end of the study, all data queries will be resolved, data cleaned and accepted before database locking. After database lock, no further entry shall be possible, and this is set for month 14.

# Handling Biological samples

## Immunological samples

Before taking any samples, correct tubes and accessories shall be checked, and the tubes labelled accordingly. Blood shall be collected through the ante-cubital vein after appropriate antiseptic procedure as stipulated in the manual of SOPs into vacutainer tubes with anticoagulant. 4 to 5mL of blood will be taken when the antibody titration, WB and ADCI. An additional 10mL will be drawn for cell mediated immunity measurement.

Each plasma sample will be divided into 1mL cryotubes and kept at –70°C until antibody responses measurement. The fresh cells will directly used for ELISPOT and the remaining cryotubes at a rate of 18-20 million cells per tube in DMSO/FCS that shall be kept in a freezer at the temperature of –80°C for two days before they are transferred into a liquid nitrogen (to be kept at -170°C). Cryotubes must be correctly labelled with the volunteer’s identification code, the date of sample collection, the day of the visit and the cryotube’s number with indelible ink.

Aliquoting must be performed subject by subject to avoid mixing blood tubes. The following procedure must be followed:

1. After centrifugation, the person responsible for aliquoting should carry out the operation by taking the tubes one by one from the centrifuge.
2. The operator will place the cryotubes in a rack before aliquoting and will affix the completed labels onto the tubes.
3. Aliquoting will be performed according to the required volume 1mL, the number of tubes, and the priority of titrations.
4. Pre-labelled Nunc tubes that have not been filled for lack of serum will be removed.
5. The subject’s identification number and initials, the date of sampling, the number of aliquots obtained, and the date and time of aliquoting will be specified on the sample identification log. Labels will be affixed onto the three sheets of the list.
6. Comments may be made on the quality of samples (haemolysed, contaminated, etc.).
7. The next sample will be taken out of the centrifuge only when the steps 1 through 6 are completed.

## Storage Conditions

The analysis of cellular immunity will be done immediately on the freshly separated cells. The plasma aliquots have to be stored at -70°C for measurement of antibodies. The remaining separated mononuclear cells from the peripheral blood will be kept in liquid nitrogen for further controls or analyses.

Storage temperatures must be monitored and documented on the appropriate form as stipulated in the manual of procedures.

## Biological evaluation samples

Two separate tubes shall be used for haematology and clinical chemistry. One containing EDTA anticoagulant shall be used for the haemogram, and another plain tube for the urea.

These tests will be done in situ at the Vaccinology unit.

Table 1: Acceptable/normal ranges for blood testing

|  | Acceptable limit/ normal range | Toxicity grading scale | | | |
| --- | --- | --- | --- | --- | --- |
|  |  | Grade 1 | Grade 2 | Grade 3 | Grade 4 |
| Hemoglobin | ≥ 8.0 g/dL | < ULN | < 6.0 g/dL | < 5.0 g/dL | < 5.0 g/dL & clinical signs of heart failure |
| Total white cell count^†^ | ≥ 4.0 x 10^3^ /μL  < 17 x 10^3^ /μL | 2.5 to 4.0 x 10^3^ /μL | 1.5 to 2.4 x 10^3^ /μL | 1.0 to 1.4 x 10^3^ /μL | < 1.0 x 10^3^ /μL |
| Platelets^†^ | ≥ 100 x 10^3^ /μL | 50 to 99 x 10^3^ /μL | 25 to 49 x 10^3^ /μL | < 25 x 10^3^ /μL | < 25 x 10^3^ /μL & clinical signs of bleeding |
| ALT* | ≤ 60 μmol/L | 1.1 to 2.5 x ULN | 2.6 to 5.0 x ULN | 5.1 to 10.0 x ULN | > 10.0 x ULN |
| Creatinine* | ≤ 60 μmol/L | 1.1 to 1.5 x ULN | 1.6 to 3.0 x ULN | 3.1 to 6.0 x ULN | > 6.0 ULN or requires dialysis |
| ^†^ Grading scale adapted from Division of AIDS table for grading severity of adult and pediatric adverse events December 2004  * Grading scale adapted from WHO Toxicity Grading Scale for Determining Severity of Adverse Events, February 2003.  ULN: Upper Limit of Normal | | | | | |

# Trial participants

## Recruitment of participants

Following authorizations from the national ethics committee, the study information will be passed on to the community of Balonghin. Starting with the local village leadership, opinion holders and other gate keepers, meetings to introduce the study programme will be organised. There will be continuous exchange of information with the community with specific question and answer sessions at the community level. Once acceptability is ascertained, parents and guardians of children aged 1-2 years will be invited to participate in the study. Adequate time will be allowed for considering the study information and those willing to participate will be invited to consent for the screening.

At screening, full medical history and physical examination will be conducted by the study clinicians.

## Inclusion criteria for enrolment

Only participants who fulfil **ALL** of the following will be enrolled in the study and allocated a study ID number.

1. Children aged 1-2 years old
2. Healthy by medical history and physical examination
3. Signed /thumb printed informed Consent by guardian/parent
4. Resident in the study area village during the whole trial period

## Exclusion criteria for enrolment

Any one of the following criteria will be exclusionary for this study:

1. Symptoms, physical signs of disease that could interfere with the interpretation of the trial results or compromising the health of the subjects.
2. Immunosuppressive therapy (steroids, immune modulators or immune suppressors) within 3 months prior recruitment. (for corticosteroids, this means prednisolone or equivalent ≥ 0.5 mg/kg/day. Inhaled and topical steroids are allowed).
3. Cannot be followed for any social, psychological or geographical reasons.
4. Use of any investigational drug or vaccine other than the study vaccine within 30 days preceding the first dose of study vaccine, or planned use up to 30 days after the third dose.
5. Suspected or known hypersensitivity to any of the vaccine components or to previous vaccine.
6. Laboratory abnormalities on screened blood samples out of range, more specifically refer to table 2.
7. Planned administration of a vaccine not foreseen by the study protocol within 30 days before the first dose of vaccine. An exception is the receipt of an EPI or licensed vaccine (measles, oral polio, meningococcal and combined diphtheria/pertussis/tetanus vaccines) which may be given 14 days or more before or after vaccination.
8. Evidence of chronic or active hepatitis B infection.
9. Presence of chronic illness that, in the judgement of the investigator, would interfere with the study outcomes or pose a threat to the participant’s health.
10. Administration of immunoglobulin and/or any blood products within the three months preceding the first dose of study vaccine or planned administration during the study period.
11. History of surgical splenectomy.
12. Moderate or severe malnutrition at screening defined as weight for age Z-score less than ‑2

## Elimination criteria during the study

The following criteria should be checked at each visit subsequent to the first visit. If any become applicable during the study, it will not require withdrawal of the subject from the study but may determine a subject’s evaluability in the according-to-protocol (ATP) analysis.

- Administration of a vaccine not foreseen by the study protocol during the period starting from 30 days before Dose 1 and ending 30 days after Dose 3.
- Use of any investigational or non-registered product (drug or vaccine) other than the study vaccines during the study period.
- Administration of immunoglobulins and/or any blood products during the study period.
- Chronic administration (defined as more than 14 days) of immunosuppressants or other immune-modifying drugs during the study period (for corticosteroids, this will mean prednisone, or equivalent, ≥ 0.5 mg/kg/day. Inhaled and topical steroids are allowed).

## Contraindications to subsequent vaccination

### Indications for deferral of vaccination

The following AEs constitute contraindications to administration of MSP3 or Hepatitis B vaccines at that point in time; if any one of these AEs occurs at the time scheduled for vaccination, the subject may be vaccinated at a later date, within the time window specified in the protocol, or withdrawn at the discretion of the investigator. AEs should be followed-up accordingly:

- Acute disease at the time of administration of investigational product (acute disease is defined as the presence of a moderate or severe illness with or without fever). All vaccines can be administered to persons with a minor illness such as diarrhea or mild upper respiratory infection without fever, i.e. axillary temperature < 37.5°C.
- Axillary temperature of ≥ 37.5°C.

### Absolute contraindications to further vaccination

The following AEs constitute absolute contraindications to further administration of MSP3 or Hepatitis B vaccines; if any of these AEs occur during the study, the subject must not receive additional doses of vaccine, but may continue other study procedures at the discretion of the investigator. AEs should be followed-up according to the protocol instructions:

- Acute allergic reaction (significant IgE-mediated events) or anaphylaxis following the administration of vaccine investigational product.
- Any confirmed or suspected immunosuppressive or immunodeficient condition, including human immunodeficiency virus (HIV) infection.

## Subject completion and drop out

## Definition of a Drop-out

From the perspective of data analysis, a 'drop-out' is any subject who is not brought back for the concluding visit foreseen in the protocol. A subject who is brought in for the concluding visit foreseen in the protocol is considered to have completed the study and is therefore not a drop-out.

## Procedures for Handling Drop-outs

Investigators should make an attempt to contact parents of those subjects who have not been brought for scheduled visits or follow-up. Information gathered should be described on the Study Completion page of the CRF and in source documents (progress notes).

## Reasons for Drop-outs

The Study Completion page of the CRF will specify which of the following possible reasons were responsible for drop-out of the subject from the study:

• Serious adverse event (SAE)

• Non-serious adverse event (AE)

• Protocol violation (Specify)

• Withdrawal of parental consent, not due to an AE or SAE

• Migration of family or subject from the study area

• Loss to follow-up

• Other (Specify)

# Investigational Products and Administration

## The MSP 3 LSP study vaccine

The anti-malaria vaccine MSP3 is a long synthetic peptide containing the amino-acid sequence 186-276. It is lyophilised with a white amorphous appearance. The vaccine is presented in multi-dose vials with the following formulation:

**Active ingredient:** MSP3/181-276. 120µg

**Excipients:**  sodium chloride 9mg/mL

Trisodium-Citrate (10mM) 2.94mg/mL)

Disodium-Phosphate buffer (10mM) 1.42mg/mL

**Manufacturer:** Synprosis Ltd

The vaccine is presented as a lyophilised powder. Before injection the vaccine is to be reconstituted in 9‰ saline solution. The reconstituted vaccine is then mixed with 1mL of aluminium hydroxide, following the procedure recommended by the manufacturer. The minimum adsorption time of 60 minutes before injection must be respected before administration of the vaccine.

It must be kept in a refrigerator between +2°C and +8°C temperature and should not be frozen. After reconstitution, the vaccine should remain useful for not more than 6 hours.

The adsorbed vaccine is then aliquoted in single-use syringes, containing 15 or 30µg of peptide. From one multi-dose vial, individual vaccine doses of 0.5mL will be prepared following SOP provided by the manufacturer.

The vaccine must be administered in the best sterile conditions, using a single-use syringe for each subject and following a strict procedure of disinfection of the injection site.

The vaccine should not be injected by the intravascular route: the investigator must check that the needle is not in a blood vessel.

As for any injectable vaccine in a phase 1 trial, it is mandatory to have available appropriate medical treatment in case of an immediate anaphylactic reaction following the administration of the vaccine.

## The Hepatitis B Control Vaccine

ENGERIX-B [Hepatitis B Vaccine (Recombinant)] is a non-infectious recombinant DNA hepatitis B vaccine developed and manufactured by GlaxoSmithKline Biologicals. It contains purified surface antigen of the virus obtained by culturing genetically engineered *Saccharomyces cerevisiae* cells, which carry the surface antigen gene of the hepatitis B virus. The surface antigen expressed in *Saccharomyces cerevisiae* cells is purified by several physicochemical steps and formulated as a suspension of the antigen adsorbed on aluminum hydroxide. The procedures used to manufacture ENGERIX-B result in a product that contains no more than 5% yeast protein. No substances of human origin are used in its manufacture.

ENGERIX-B is supplied as a sterile suspension for intramuscular administration. The vaccine is ready for use without reconstitution; it must be shaken before administration since a fine white deposit with a clear colourless supernatant may form on storage.

For the blinding purpose, the vaccine will be given at a schedule of 0,1 and 2 months, subcutaneously. Subcutaneous administration may result in lower anti-HBs antibodies GMT; however, a blood sample will be taken at 6 month post dose one to identify children who do not reach sero-protective level of antibodies. They will be revaccinated at the same time with the MSP3 vaccinated at the end of the study.

# Vaccine administration

All enrolled subjects shall receive three injections by the sub-cutaneous route at days 0, 28 and 56. The vaccination site will be the external side of the arm, at the junction of the superior third and inferior two-thirds. The first and third injections will be given on the left arm, the second injection in the right arm.

## Prior and Concomitant Therapy

Immunosuppressive or immune-modulator treatments are non-inclusion criteria. If it happens to be necessary to use one of them during the trial period, the data for the concerned participant will be considered as a deviation to the protocol.

Antipyretics or painkillers will not be allowed as preventive treatment of pain or fever, before vaccination. Concomitant vaccinations are not allowed during the study; an exception is the receipt of an EPI or licensed vaccine (measles, oral polio, meningococcal and combined diphtheria/pertussis/tetanus vaccines) which may be given 14 days or more before or after vaccination.

For all other treatments, the trade name of the drug, total daily dose, indication, start and stop dates will be required for the CRF completion (and, if appropriate, timing requirements within the day: AM, PM, post-vaccination shot)

Table 2 Summary of time periods between which different classes of concomitant medication/treatment/vaccination must be recorded

| **3 months prior to Dose 1 → Dose 1** | All treatments listed as elimination criteria in Section 8.48.4 |
| --- | --- |
| **Screening → 30 Days post Dose 3** | All antipyretic, analgesic, antibiotic and any treatments listed as elimination criteria in Section 8.4  All vaccinations |
| **31 Days post Dose 3 → Final Study Visit** | All treatments listed as elimination criteria in Section 8.4 |
|  | |

## Management of Vaccines

### Labelling and Packaging

Vaccines will be packaged in multi-dose vials with labels indicating that this vaccine is for clinical study use only.

Label for group 1

| **Study Code: MSP3_BF_0302**  MSP3 - 120µg  8 subcutaneous doses  Keep between 2º and 8ºC . **Do not Freeze**  Discard if frozen  Please contact: Dr Sirima Tel : +226 70 200 444  ***Dose for clinical Use only***   | Vial n°:……….. | | --- |   African Malaria Network Trust |
| --- | --- |

**Label for group 2**

| **Study Code: MSP3_BF_0302**  MSP3 - 120µg  4 subcutaneous doses  Keep between 2º and 8ºC. **Do not Freeze**  Discard if frozen  Please contact: Dr Sirima Tel : +226 70 200 444  Dose for clinical Use only   | Vial n°:……….. | | --- |   African Malaria Network Trust |
| --- | --- |

### Storage and Shipment Conditions

The pharmacist will be responsible for the study’s vaccine management. However, he may name another person to undertake this function, the latter being directly under his responsibility.

AMANET is responsible for provision of appropriate labelling and packaging. Vaccines will be shipped along with a delivery voucher from the Henogen in Belgium to the CNRFP, maintaining the +2 to 8^o^C cold chain and the international regulations according to a schedule pre-established with the investigator. After reception of the vaccines at the CNRFP, the delivery voucher will be dated and signed by the pharmacist and a copy will be sent to AMANET. The receipt of vaccines will be registered in a document that should mention the batch numbers, dosage, and the number of doses per batch together with the manufacture, receipt and expiry dates. This information will be kept at AMANET.

The vaccines will be kept between +2°C and +8°C, in a refrigerator whose use is be reserved for vaccines only, under controlled access, with a continuous rotary disk internal temperature recorder and an alarm warning of any break in the cold chain. The temperature will be checked physically and recorded twice a day on the external record of the refrigerator. Periodically recorded temperatures will be registered in a special document covering the whole study period. The temperature recording disks will be noted down daily, and archived in as essential documents. Transportation of needed vaccine vials to the Vaccinology unit in Balonghin will be under strict SOPs just before use. Temperature recorders will be placed in the cool boxes to ensure that temperatures during transportation were maintained at acceptable levels.

In case of a cold chain break, endangering degradation of vaccine antigens, a temperature deviation form will be filled in to notify the sponsor and the manufacturer. Final decision to continue the use of the vaccines will remain with the sponsor.

### Accountability

The vaccines’ traceability will be ensured at all times. At each vaccination session the dose given to each subject will be recorded. AMANET will ensure that adequate vaccines are supplied at the trial sites. A vaccine accountability log book will be routinely checked during each monitoring visit against the trial participants receiving vaccines. Any discrepancy has to be well documented and justified.

An overall inventory will be undertaken at the end of the study and any unused vaccines will be sent back to AMANET.

Adequate supplies of the Hepatitis B vaccine will also be supplied to cover for cross over vaccination of participants who would have received the malaria vaccine at the end of the study when the trial is “unblinded”.

### Return of Unused Products

Unused unsealed products will be sent back to AMANET at the end of the vaccinations labelled “unsealed or unused product return”. This will be after the study monitor has verified vaccine accountability. All used vials empty vials must also be returned.

# Adverse events management and reporting

## Definitions

### Adverse Event (or Adverse Experience)

Any untoward medical occurrence in a patient or clinical investigation participant administered a pharmaceutical product and which does not necessarily have to have a causal relationship with this treatment.

An adverse event (AE) can therefore be any unfavourable and unintended sign (including an abnormal laboratory finding), symptom, or disease temporally associated with the use of a medicinal product, whether or not considered related to the medicinal product.

Examples of an AE include:

• Exacerbation of a chronic or intermittent pre-existing condition including either an increase in frequency and/or intensity of the condition.

• New conditions detected or diagnosed after investigational product administration even though it may have been present prior to the start of the study.

• Signs, symptoms, or the clinical sequelae of a suspected overdose of either investigational product or a concurrent medication (overdose per se should not be reported as an AE/SAE).

• Signs, symptoms temporally associated with vaccine administration.

AEs may include pre- or post-treatment events that occur as a result of protocol-mandated procedures (i.e., invasive procedures, modification of subject’s previous therapeutic regimen).

### Serious Adverse Event

During clinical investigations, adverse events may occur which, if suspected to be medicinal product-related (adverse drug reactions), might be significant enough to lead to important changes in the way the medicinal product is developed (e.g., change in dose, population, needed monitoring, consent forms). This is particularly true for reactions which, in their most severe forms, threaten life or function. Such reactions should be reported promptly to regulators.

Therefore, special medical or administrative criteria are needed to define reactions that, either due to their nature ("serious") or due to the significant, unexpected information they provide, justify expedited reporting. To ensure no confusion or misunderstanding of the difference between the terms "serious" and "severe," which are not synonymous, the following note of clarification is provided:

The term "severe" is often used to describe the intensity (severity) of a specific event (as in mild, moderate, or severe myocardial infarction); the event itself, however, may be of relatively minor medical significance (such as severe headache). This is not the same as "serious," which is based on patient/event outcome or action criteria usually associated with events that pose a threat to a patient's life or functioning. Seriousness (not severity) serves as a guide for defining regulatory reporting obligations

A serious adverse event (experience) or reaction is any untoward medical occurrence that at any dose:

- - results in death,
  - is life-threatening,

NOTE*:* The term "life-threatening" in the definition of "serious" refers to an event in which the patient was at risk of death at the time of the event; it does not refer to an event which hypothetically might have caused death if it were more severe.

- - requires inpatient hospitalisation or prolongation of existing hospitalisation,

*NOTE: In general, hospitalization signifies that the subject has been detained (usually involving at least an overnight stay) at the hospital or emergency ward for observation and/or treatment that would not have been appropriate in the physician’s office or out-patient setting. Complications that occur during hospitalization are AEs. If a complication prolongs hospitalization or fulfills any other serious criteria, the event is serious. When in doubt as to whether ‘hospitalization’ occurred or was necessary, the AE should be considered serious.*

*Hospitalization for elective treatment of a pre-existing condition that did not worsen from baseline is not considered an AE.*

- - results in persistent or significant disability/incapacity, or

*NOTE: The term disability means a substantial disruption of a person’s ability to conduct normal life functions. This definition is not intended to include experiences of relatively minor medical significance such as uncomplicated headache, nausea, vomiting, diarrhea, influenza, and accidental trauma (e.g. sprained ankle) which may interfere or prevent everyday life functions but do not constitute a substantial disruption.*

- - is a congenital anomaly/birth defect.

Medical and scientific judgement should be exercised in deciding whether expedited reporting is appropriate in other situations, such as important medical events that may not be immediately life-threatening or result in death or hospitalisation but may jeopardise the patient or may require intervention to prevent one of the other outcomes listed in the definition above. *These should also usually be considered serious.*

## Clinical laboratory parameters and other abnormal assessments qualifying as adverse events and serious adverse events

Abnormal laboratory findings (e.g., clinical chemistry, hematology, urinalysis) or other abnormal assessments (e.g. blood film ) that are judged by the investigator to be clinically significant will be recorded as AEs or SAEs if they meet the definition of an AE, as defined in Section 11.1.1 or SAE, as defined in Section 11.1.2. Clinically significant abnormal laboratory findings or other abnormal assessments that are detected during the study or are present at baseline and significantly worsen following the start of the study will be reported as AEs or SAEs.

The investigator will exercise his medical and scientific judgment in deciding whether an abnormal laboratory finding or other abnormal assessment is clinically significant.

## Management of Adverse events

The investigators shall make every effort to detect all AE and SAE’s occurring in this study. Further, the events must be followed up to an established outcome of the event. The investigators will provide the best treatment available in Burkina Faso for the study participants who may require such treatments.

## Safety Data Collection and Reporting

### Expected Adverse Vaccine Reactions

As with any adjuvanted vaccine when administered subcutaneously, local reactions are expected. Systemic reactions are less often observed. Standardized data collection of adverse reactions will be organized as per AMANET ADR form.

The previous experience of MSP3 in study volunteers identified the following local reactions which should be monitored: pain, erythema, induration, pruritus, and swelling. There is a lack of information concerning expected systemic reactions; therefore, special attention will be given to general signs, i.e. fever, headache, malaise, fatigue, drowsiness, as well as signs of allergic reactions, i.e. urticaria, oedema, contra-lateral reactions, diffuse erythema.

**Solicited Local (injection site) adverse events**

- Pain at injection site
- Swelling at injection site
- Induration at injection site
- Erythema at injection site
- Pruritus at injection

**Solicited systemic adverse events**

- Fever (defined as axillary temperature ≥ 37.5°C)
- Drowsiness
- Loss of appetite
- Irritability/fussiness

### Safety Data Collection

All the adverse events/reactions, whether observed by the investigator or reported by the subject, will be carefully and accurately documented in the case report form by the investigator. For each event/reaction the following details will be recorded: 1) description of the event(s)/reaction(s), 2) date and time of occurrence, 3) duration, 4) intensity, 5)assessment of relationship to the vaccine, 6) action taken including treatment, 7) outcome.

### Collection and follow-up of adverse events

Adverse events will be recorded as indicated in the CRF. .

Adverse events likely to be related to the product (or to the trial), whether serious or not, which persist at the end of the trial will be followed up by the investigator until their complete disappearance. The investigator will inform the Monitor of the date of final disappearance of the adverse event and will document it on a correction sheet.

Moreover, any serious adverse event likely to be related to the product and occurring after trial termination should be reported by the investigator to AMANET according to the procedure described below. The active monitoring of SAES will end after the last visit of the last subject. However, SAEs occurring after the closure of the trial could passively be collected either during the epidemiological surveillance or by the health centre personnel.

### Assessment of intensity

Intensity of the following AEs will be assessed as described:

Intensity scales for solicited symptoms in infants/toddlers and children less than 6 years of age

| **Adverse Event** | **Intensity grade** | **Parameter** |
| --- | --- | --- |
| Pain at injection site | 0 | Absent |
|  | 1 | Minor reaction to touch |
|  | 2 | Cries/protests on touch |
|  | 3 | Cries when limb is moved/spontaneously painful |
| Swelling at injection site | | Record greatest surface diameter in mm |
| Induration at injection site |  | Record greatest surface diameter in mm |
| Erythema at injection site |  | Record greatest surface diameter in mm |
| Pruritus at injection site | 0 | Absent |
|  | 1 | Easily tolerated by the subject, causing minimal discomfort and not interfering with everyday activities. |
|  | 2 | Sufficiently discomforting to interfere with normal everyday activities. |
|  | 3 | Prevents normal, everyday activities. |
| Fever* |  | Record temperature in °C |
| Irritability/Fussiness | 0 | Behavior as usual |
|  | 1 | Crying more than usual/ no effect on normal activity |
|  | 2 | Crying more than usual/ interferes with normal activity |
|  | 3 | Crying that cannot be comforted/ prevents normal activity |
| Drowsiness | 0 | Behavior as usual |
|  | 1 | Drowsiness easily tolerated |
|  | 2 | Drowsiness that interferes with normal activity |
|  | 3 | Drowsiness that prevents normal activity |
| Loss of appetite | 0 | Appetite as usual |
|  | 1 | Eating less than usual/ no effect on normal activity |
|  | 2 | Eating less than usual/ interferes with normal activity |
|  | 3 | Not eating at all |
| *Fever is defined as axillary temperature ≥ 37.5°C | | |

The maximum intensity of local injection site swelling, induration and erythema will be scored as follows:

| 0 | None |
| --- | --- |
| 1 | < 5 mm |
| 2 | 5 to 20 mm |
| 3 | > 20 mm |

The maximum intensity of fever will be scored as follows:

| 0 | < 37.5°C |
| --- | --- |
| 1 | 37.5 – 38.0°C |
| 2 | > 38 – 39.0°C |
| 3 | > 39.0°C |
|  |  |

### Assessment of causal relationship

The causal relationship between the SAE and the product will first be evaluated by the investigator using the following scale:

**0.** **NO RELATIONSHIP**: Inconsistent temporal relationship (too long interval between injection and onset of symptoms or symptoms appeared before injection) or evidence that symptoms are definitely related to an aetiology other than the study treatment.

**1.** **POSSIBLE**: Has a temporal relationship with the study treatment; however, a potential alternative aetiology which may be responsible for the symptom has not been investigated.

**2.** **PROBABLE**: Has a **relevant** temporal relationship to the study treatment, a suggestive symptomatology and a potential alternative aetiology is not apparent.

**3. DEFINITE**: Has a **relevant** temporal relationship to the study treatment and no alternative aetiology is apparent (after investigation other aetiologies have been ruled out) or positive rechallenge with a suggestive symptomatology or local reaction at the site of injection.

Then, according to the available information and the current medical knowledge, the Sponsor will also assess the causal relationship to the product.

The decision to modify or discontinue the trial, or all study codes may be made after mutual agreement between AMANET and the investigator(s) and the DSMB.

### Assessment of Outcome

After the initial AE/SAE report, the investigator is required to proactively follow each subject and provide further information to AMANET on the subject’s condition.

All AEs and SAEs documented at a previous visit/contact and designated as not recovered/not resolved or recovering/resolving will be reviewed at subsequent visits/contacts.

Investigators will follow-up subjects:

- with SAEs or subjects withdrawn from the study as a result of an AE, until the event has resolved, subsided, stabilized, disappeared, the event is otherwise explained, or the subject is lost to follow-up;
- or, in the case of other non-serious AEs, until they complete the study or they are lost to follow-up.

All Grade 3, Grade 4 or clinically significant laboratory abnormalities will be followed up until they have returned to normal, or a satisfactory explanation has been provided. Additional information (including but not limited to laboratory results) relative to the subsequent course of such an abnormality noted for any subject must be made available to the Study Monitor.

AMANET may request that the investigator perform or arrange for the conduct of supplemental measurements and/or evaluations to elucidate as fully as possible the nature and/or causality of the AE or SAE. The investigator is obliged to assist. If a subject dies during participation in the study or during a recognized follow-up period, AMANET will be provided with a copy of any available post-mortem findings, including histopathology.

New or updated information will be recorded on the originally completed SAE Report Form, with all changes signed and dated by the investigator. The updated SAE report form should be resent to AMANET within 24 hours of receipt of the follow-up information.

Outcome of any non-serious AE occurring within 30 days post-vaccination (i.e. unsolicited AE) or any SAE reported during the entire study will be assessed as:

- Recovered/resolved
- Not recovered/not resolved
- Recovering/resolving
- Recovered with sequelae/resolved with sequelae
- Fatal (SAEs only).

### Reporting of Serious Adverse Events

Every serious adverse event occurring throughout the trial must be notified to the AMANET by the investigator as **soon as he is alerted to it**, i.e., **within 24 hours of being alerted**, even if the investigator considers that the adverse event is not related to treatment. The dully completed SAE form should be submitted **within 5 working days**, and the same must also be transmitted to the chair of the DSMB bearing nor personal identifiers or treatment allocation.

Any relevant information concerning the adverse event that becomes available after the SAE report form has been sent (outcome, precise description of medical history, results of the investigation, copy of hospitalisation report, etc.) should be forwarded as soon as possible to the Sponsor and the chair of the DSMB within 24 hours from the investigator learning of it. The anonymity of the subjects shall be respected when forwarding this information.

| **Study Contact for Reporting of a Serious Adverse Event** | |
| --- | --- |
| All three of the contacts listed below must be informed of each SAE | |
| **Study Contact at AMANET for Reporting Serious Adverse Events** | |
| **Roma Chilengi**, BSc. MB ChB, Clin Research, DHTM  Clinical Trials Coordinator,  Tanzania Commission for Science and Technology Building Building,  P.O. Box 33207  Dar es salaam, Tanzania  Tel: (255) 22 2700018 Fax:  255 (0)22 2700380  E-mail: [chilengi@amanet-trust.org](mailto:chilengi@amanet-trust.org) | |
| **Local safety monitor** | **Clinical Monitor** |
| To be named | **Mahamadou Aly THERA,** MD, MPH  Malaria Research and Training Center (MRTC)  Department of Epidemiology of Parasitic Diseases (DEAP) Faculty of Medicine, Pharmacy and Dentistry, (FMPOS)  University of Mali; BP: 1805 Bamako |
|  | |

## Regulatory requirements

In order to comply with current regulations on serious adverse event reporting to Health Authorities the investigator shall document accurately the event, respect notification deadlines, provide AMANET with all necessary information and, if requested, to give access to source documents.

The Investigator shall further inform the relevant Health Authorities as soon as possible, of any serious adverse event likely to be related to the product (or to the trial). AMANET shall also inform the Authorities of any trial discontinuation and specify the reason for discontinuation.

The principal investigator will inform the other investigators and the Ethics Committee of the occurrence of any serious adverse drug reaction.

## Blood sampling

A blood sample, to be taken as soon as possible, may be requested in case of a serious adverse event if it might help in determining the cause of the SAE. An appropriate blood volume in a correct container would be obtained for relevant tests.

## Lost to Follow-up Procedures

If a participant fails to appear for a follow-up examination, intensive efforts to visit them at home will be undertaken to recall them or at least to determine their health status. These efforts will be documented in the subject’s CRF and source documents.

## Specific case: Diagnosis and treatment of Malaria cases

### Diagnosis

Although symptoms of uncomplicated malaria can be rather non specific, in this study, malaria diagnosis should be confirmed by detection of parasitaemia. However, in high transmission areas such as Balonghin, where the immunity is high, asymptomatic parasitaemia is common. The following clinical signs significantly associated to malaria have been observed and are widely acceptable for suspicion of malaria:

(a) Axillary temperature ≥37.5° C;

(b) Axillary temperature < 37.5° C, but with history of fever within the preceding 24 hours;

(c) History of illness with other signs than fever,

Patients presenting with these features will be investigated for malaria, and the decision to provide treatment will be at the discretion of the clinical investigators.

### Malaria case management

- **Uncomplicated Malaria Cases**

Any participant diagnosed as an uncomplicated malaria case will receive Coartem® 20/10mg (artemether 20mg-lumefantrine 120mg) using a 4-dose regimen (16 tablets, 320 mg artemether and 1920 mg lumefantrine, given over 2 days) as oral first-line treatment. Therapy is delivered over 2 days with the dosage based on the patient’s body weigh; detailed instructions on dosing is provided in the manual of SOP under treatment of malaria at time 0, 8, 24 and 48 hours.

- **Severe Malaria Cases**

The treatment is based on quinine salts delivered by IV slow perfusion as follows:

- A loading dose of 20 mg dihydrochloride salt/kg of body weight (loading dose) 1 diluted in 10 ml isotonic fluid/kg by IV infusion over 4 hours;
- then 8 hours after the start of the loading dose, give a maintenance dose of quinine, 10 mg salt/kg, over 4 hours. This maintenance dose should be repeated every 8 hours, calculated from the beginning of the previous infusion, until the patient can swallow,
- then Coartem at the same dosage regimen as for uncomplicated malaria.

Response should be monitored by frequent clinical examination including recording of fluid balance, temperature, pulse, respiratory rate and depth, level of consciousness, blood pressure, jugular venous pressure, and parasitaemia (in blood films) every 4–6 hours for the first 48 hours.

# Primary evaluation criteria

## Definition of the Criterion

The reactogenicity and the safety shall be assessed on the following criteria:

1. Immediate reactogenicity (within 60 minutes, with emphasis on allergic reactions),
2. Local and systemic reactogenicity during the 7 days following each vaccination.
3. Any unsolicited adverse events (AE) occurring outside scheduled visit times, up to one month after the third vaccine dose and requiring a physician visit
4. Any serious adverse events (SAE) whenever they occur during the trial period (from first visit to last visit). The relationship with the vaccine must be determined by the investigator, using the following definitions: **not related, possibly related, probably related, definitely related.**
5. Biological safety, assessed four weeks after each injection, in relation to the baseline data profile on the following:

RBC, haemoglobin, haematocrit, platelets, WBC, Liver enzymes (SGOT, GPT, total bilirubin) and creatinine.

## Parameters to be measured

The rate, duration and severity of signs and symptoms during the 7 days after each vaccination will be measured.

## Solicited symptoms

### Local reactions

The following local reactions will be solicited immediately after the injection (60 minutes) and over the 7 days following the vaccination (D1 and D7 follow-up) at the site of injection (left arm for 1st and 3rd injection; right arm for the 2nd injection) :

**Any local**

- 1. Pain at injection site
  2. Swelling at injection site
  3. Induration at injection site
  4. Erythema at injection site
  5. Pruritus at injection

### Systemic reactions

The following systemic reactions will be systematically solicited immediately after the injection (60 minutes) and during the 6 days following the vaccination (D1 to D6 follow-up).

**Any systemic**

1. Fever (defined as axillary temperature ≥ 37.5°C)
2. Drowsiness
3. Loss of appetite
4. Irritability/fussiness

## Unsolicited symptoms

Unsolicited symptom is defined as any adverse event (AE) reported in addition to those solicited during the clinical study. Also any “solicited” symptom with onset outside the specified period of follow-up for solicited symptoms will be reported as an unsolicited adverse event.

Unsolicited symptoms will be recorded from the first dose of vaccination to 30 days post third dose.

For the unsolicited symptoms, intensity will be assessed as follows:

| Mild | Easily tolerated by the subject, causing minimal discomfort and not interfering with everyday activities. |
| --- | --- |
| Moderate | Sufficiently discomforting to interfere with normal everyday activities. |
| Severe | Prevents normal, everyday activities. |

Outcome and causal relationship will be assess as described in section 13.4.2.3 and 13.4.2.4

# Secondary Evaluation Criteria: Immunogenicity

## Humoral Immune response

### Definition of the Criteria

The humoral response to vaccine antigens will be assessed by measuring by ELISA on D0, D28, D56, D84, D168 and D365, the levels of:

- IgG, (peptide et epitopes A, B, C and D)
- Isotypes IgG1, IgG2, IgG3, IgG4, (peptide)
- IgM (peptide)

### Parameters to be measured

The levels of IgG and IgM will be expressed as concentration (ng/ml). The sero-response will be defined in the post-vaccination to re-vaccination ratio. (D28/D0, D56/D0, D84/D0, D168/D0, and D365/D0).

Because of the prior natural exposure of trial subjects to Plasmodium falciparum and so to MSP3 antigen, it is anticipated that almost all participants will be sero-positive before vaccination occurs. Consequently, the sero-conversion rate, defined as the number of pre-vaccination sero-negative candidates having a concentration considered as positive after vaccination, will not be used.

### Method and Timing of Measurement

IgG concentrations against these antigens will be measured by ELISA (Enzyme Linked Immuno Sorbent Assay), which is an immuno-enzymatic technique that allows levels of blood antibodies to be measured.

Levels of IgG, IgM and isotypes IgG1, IgG2, IgG3 and IgG4 will be measured in the blood samples collected at visits V1, V17, V32, V33, V48, V49 and V50 i.e. on days D0, D28, D56, D112, D140, D252 and D365.

## Cellular Immune response

### Definition of the Criteria

The cellular immune response to vaccine’s antigens (peptide) will be assessed by measuring

The number of cells producing IFNγ by Elispot to MSP3-LSP and peptides MSP3 a,b,c,d before and four weeks after the second and the third injection.

### Method and timing

The ELISPOT will be done after in vitro stimulation with the peptide or peptide fragments (A, B, C and D) of peripheral blood mononuclear cells. The spots will be counted using ELISPOT reader.

The cells producing IFN-γ will be measured before (D0) and one month after the second (D56) and one month after the last injection (D84).

### Parameters to be measured

The cells producing IFNγ by Elispot expressed in number of spots /10^6^ cells

## Exploratory assays: Functionality of immune response

The following exploratory tests shall be conducted to assess the functionality and quality of the induced immune responses:

### Native protein recognition on Merozoite

Mature schizont-stage proteins of *P. falciparum* (3D7 clone) will be extracted in Laemmli sample buffer, submitted to electrophoresis, and transferred to nitrocellulose as described^9^ Nitrocellulose strips will then be incubated with each sera and the reaction revealed.

The intensity of the reactivity was classified as negative, positive (+), or strongly positive (++).

### Antibody Dependant Cytophilic Inhibition

The functionality of IgG shall be assessed by using the Antibody Dependant Cytophilic Inhibition (ADCI) technique.

The assays for assessing functionality of immune responses will be done on samples obtained on the following days: D0, D84, D168 and D365

# Statistical Methods and Data Analysis

## Principal Objective

To assess the safety and reactogenicity of 3 doses of 30 µg and 15 µg MSP3 adjuvanted in aluminium hydroxide given at D0, D28 and D56 in healthy children aged 4-6 and 1-2 years old in Burkina Faso.

This will be achieved through conducting a randomised controlled trial. The trial will certainly be single blinded to the participants. Efforts will be made towards a double blind design in that concealment of vaccination will be ensured to all who will be involved in the objective assessment of trial endpoints.

## Sample size considerations

The trial sample size will be determined on the primary evaluation criteria, concerning the occurrence of systemic adverse events. The total sample size is calculated at 45 children to be distributed as shown in the table below:

| 1-2 Year Olds |
| --- |
| 15 receiving 15µg MSP 3 vaccine |
| 15 receiving 30µg MSP 3 vaccine |
| 15 receiving Hepatitis B vaccine |
| **45 Children in total** |

The hypothesis in absolute terms has the following assumptions considered:

The lost of follow-up or protocol deviation is anticipated to be no more than 10%. Therefore with a total sample size of 45, the study is adequately powered to show such a difference if the assumptions hold.

With 14 children completing follow-up in each MSP3 arm, the study will be have 90% power to detect at least one MSP3 vaccinated individual with a systemic reaction (or a serious adverse event) if the underlying risk of such an event is 15% or more. (The trial will have 80% power to detect at least one individual if the underlying risk is 11% or more and 95% power to detect at least one individual if the underlying risk is 19% or more).

## Data Set to be analysed

### Definition of Population

### Total Vaccinated Cohort

The Total Vaccinated Cohort will include all vaccinated subjects for whom data are available. Thus, the total analysis of safety will include all subjects with at least one vaccine administration documented and the total analysis of immunogenicity will include vaccinated subjects for whom data concerning immunogenicity endpoint measures are available. The Total Vaccinated Cohort analysis will be performed per treatment actually administered.

### According to protocol (ATP) cohort for analysis of safety

The ATP cohort for analysis of safety will include all evaluable subjects;

- who have received at least one dose of study vaccine according to their random assignment
- have sufficient data to perform an analysis of safety (at least one vaccine dose with safety follow-up)
- for whom administration site of study vaccine is per protocol
- who have not received a vaccine not specified or forbidden in the protocol and for whom elimination criteria were not applied
- for whom the randomization code has not been broken except for when unblinding has been carried out by the DSMB for Safety Analysis.
- who meet all eligibility criteria.

### According to protocol (ATP) cohort for analysis of immunogenicity

The ATP cohort for analysis of immunogenicity will include all evaluable subjects (i.e. those meeting all eligibility criteria, complying with the procedures defined in the protocol, with no elimination criteria during the study) for whom data concerning immunogenicity endpoint measures are available.

For a given subject and a given immunogenicity measurement, missing or non-evaluable measurements will not be replaced. Therefore, an analysis will exclude subjects with missing or non-evaluable measurements.

## Statistical Methods

The analysis shall be descriptive; the sample size does not allow any comparison between groups. For categorical variables, frequency distributions, by vaccination group, will be presented. For continuous variables, box-whisker plots, medians, inter-quartile ranges and ranges will be presented by vaccination group.

## Data Management

The data management will be performed at CNRFP. The data base will be developed in Microsoft Access software®. The specific procedures for validation, cleaning, and locking the data have been detailed in the study SOP’s on data management.

The data entry will be performed in real time as it is generated. However, the senior investigators have to sign off all CRF pages before being taken for data entry. The source documents have been described on the official AMANET source document declaration form.

For each data series, procedure for double entry, quality control and coherence queries procedures will be implemented systematically in order to detect errors and/or missing data. After integration of all the data corrections on the whole data base, the data base will be locked and saved before to be transferred to the trial statistician. Each step of the process will be controlled by implementation of individual passwords and/or regular back-ups, in order to allow access to the data base and to ensure its integrity.

# Ethical Considerations

## Informed Consent

The participant’s parents and guardians will be informed of the trial’s goals, of the methodology, and of potential risks. Each parent or guardian will be able to discuss with the principal investigator or study physician and will have opportunity to ask any question. They will be allowed a three-day period to discuss and be informed by a qualified person before signing any consent file.

Before blood sampling, all the children and their parents will be informed of what tests the sampling is for. Adequate preparations have been made to ensure asepsis in the process. However, any untoward event following a study procedure will be given the best possible treatment in Burkina Faso.

Ms Assétou Dermé, a qualified behavioural scientist at CNRFP, will be responsible of the process of informed consent.

## Risks and benefits

There will be no direct benefit for the participants arising from receiving the test vaccine as the knowledge about MSP3 vaccine is yet limited to extrapolate its effects in the trial population. Parents and guardians will be informed especially about the trial’s constraints (number of injections, consultations, blood tests, etc) and the strict confidentiality of the collected data as well as the possibility to withdraw at any moment, their participation consent without need for justification. A written and witnessed (if illiterate) consent will be mandatory. In case of withdrawal after the first vaccination, the concerned participant will still have access to the same level of the heath care as others remaining in the trial.

All the children participating in this trial would not have publicly received vaccination against Hepatitis B. This vaccine has recently been adopted into the EPI programme by the Burkina Faso Ministry of Health. Therefore the children in the control arm will benefit from this vaccine. At the end of the study when vaccine allocation is revealed, all the children who received the MSP 3 vaccine will also be immunised against Hepatitis B through this study.

All of the expenses related to participating in this such as transportation, meals on vaccination sessions or to medical care (evacuation, disease, accident etc) will be supported by the trial during the whole trial period.

The ultimate justification of this study lies in the possibility of developing a vaccine that protects children against malaria. The community in Balonghin will ultimately benefit through their national Health and EPI programme should this vaccine lead to a registered product. Such a benefit will in fact be extended to the entire country and malaria endemic regions.

## Compensation

The participants and their parents or guardians will be granted compensation for the loss due to participation in this i.e. lost day’s work, transportation to scheduled visits etc. Transport will be provided for all the scheduled visits, and costs will be reimbursed for any other unscheduled visits. Compensation for lost days of work will be made in kind. Each volunteer will get supplies (rice, beans, oil, sugar and salt) equivalent to the lost workdays. The daily amount of supplies to serve will be set on the International Funds for Food basis i.e. 500g of rice, 80g of beans, oil, salt and sugar. We consider it a lost working day, all days when a contact with the volunteer is necessary (sorting out days, vaccination days, post vaccine follow-up days).

AMANET has a trial insurance policy though which appropriate compensation would be accorded in case of trial related events or disability. Any participant experiencing an event related to the trial that causes such disability will be eligible to claim compensation. The financial compensation for temporary or definitive after-effects or death cases will be covered by the insurance company STRATGIS Tanzania Limited.

## Ethical review and approval

Before the study can start, this protocol would have been approved by the National ethic committee of Burkiba Faso. The principal investigator will submit the trial protocol and its annexes to the Ethics Committee for Health Research. Only after receiving the original signed unconditional approval will AMANET authorise the start of the trial and ship the study vaccines. Further, efforts will be made to ensure approval of the local village chief and headmen.

Moreover, this clinical trial will be carried out according to the ethic rule set by the Helsinki Declaration (revised in Scotland and amended in Washington in 2002).

# Quality assurance and quality control

SOPs for quality management have been developed, and will be used to train appropriate personnel, and kept on file with documentation of training. Data will be evaluated for compliance with protocol and accuracy in relation to source documents. The study will be conducted in accordance with procedures identified in the protocol. The types of materials to be reviewed, who is responsible, and the schedule for reviews will be referenced in the SOPs. Study-specific training will be provided for all staff prior to the commencement of the trial.

## Access to documents

SOPs will be used at all clinical and laboratory procedures. Regular monitoring will be performed according to GCP/ICH (e.g., procedures, data and ethics compliance). The monitor will undertake 100% source document verification and report in accordance with AMANET procedures.

Direct access to all trial related documents; source data, SOP’s, case files CRF’s etc will be ensured for the purpose of monitoring and auditing by the sponsor, and inspection by local and regulatory authorities.

## Study personnel

Only appropriate persons qualified by training and experience will be responsible for study procedures. The study personnel file will maintain up to date cv’s of all staff which will be in the investigator file as part of the essential documents.

Only authorised staff will be allowed to write on study document. An authorised signatory list will also be maintained in the investigator file.

## Changes to Protocol

No amendments to this protocol will be made without consultation and expressly written agreement of AMANET. Any amendment to the trial that appear necessary during the course of the trial must be discussed and approved before it becomes effective. An amendment with changes in the participant risk benefit ratio requires IRB approval.

All amendments must also be transmitted to Regulatory Authorities, if applicable.

An administrative change to the protocol is defined as one which only modifies administrative and logistical aspects of a protocol but does not affect the subjects’ safety, the objectives of the trial and its progress. Such an administrative change does not require IRB. However, IRB must be notified whenever an administrative change is made.

The investigator is responsible for insuring that changes to an approved trial, during the period for which IRB approval has already been given, is not initiated without IRBs review and approval except to eliminate apparent immediate hazards to the subject.

## Investigational procedures

The following standard operating procedures (SOP) are shall be applied by the study team with the overall responsibility and supervision of the principal investigator:

1. SOP of the visits

- - SOP of the selection visit
  - SOP of the vaccination and immediate post vaccine follow-up visits
  - SOP of the daily follow-up visits after vaccination
  - SOP of the Blood sampling visit
  - SOP of the closing visit

2. SOP of the MSP3 and Hepatitis B vaccines preparation

3. SOP of the communication with the Ethics Committee

4. SOP of the investigator file constitution (including classification and archiving)

5. SOP for obtaining the informed consent

6. SOP of the protocol compliance

7. SOP of the medical care of the volunteers

8. SOP on the recording of Adverse Events

9. SOP on the recording and notification of Serious Adverse Events

10. SOP on the vaccine storage and accountability

11. SOP on the samples storage and accountability

12. SOP on the premature termination or suspension of the trial

13. SOP on the interim and final reports

## Monitoring

### Pre-trial and study initiation visits

A pre-trial set-up visit will be performed following the AMANET SOP. During this visit, a clear understanding of the protocol will be reached with the investigational team. Practical implications of all protocol procedures will be evaluated and rehearsed at least one month before prior to study start.

The study monitor will ensure that all designated staff understand their SOP and all requirements are in place. A trial specific training and orientation to GCP will be undertaken. The pre-trial visit report will be submitted for approval according to AMANET procedures before the initiation of the study can be allowed.

During the trial initiation visit, all problems identified during the pre-trial visit must have been resolved. The Monitor will verify and document that the materials to be used during the trial have been received and that the investigational team has been properly informed about the trial, regulatory requirements, and the AMANET SOPs.

### Routine monitoring visits

Regular routine monitoring visits will be set up and agreed with the principal investigator. This must include a pre-trial, initiation, during each vaccination, once during the long term safety follow up and a study closure visit at the end of the study.

The monitor’s duty will include, but is not limited to the following:

- carry out a quality control of trial progress: respect of protocol and operating guidelines, data collection, signature of consent forms, completion of document and appearance of SAE,
- sample and product management, cold chain monitoring,
- collect the CRFs and correspondent correction sheets,
- assess the inclusions in order to evaluate the number of complete or on-going observations.

The monitor will check that the study is progressing according to protocol. Any deviations will be reported and appropriate solutions proposed for any problems observed.

### Study close out visit

A close-out visit will be performed at the end of the trial. The objectives of this visit will be to ensure that:

- the center has all the documents necessary for archiving,
- all samples have been shipped,
- all unused material has been recovered,
- all products have been returned to the sponsor.

## Audits

The investigator shall keep all trial documents provided by AMANET for at least 15 Years after the completion or discontinuation, whatever the nature of the investigational Centre (private practice, hospital, institution).

Should there be any change in address; the principal investigator will inform AMANET in writing of that address change.

A systematic and independent examination of trial related activities and documents to determine whether this trial is conducted, recorded, analysed and accurately reported according to protocol, SOP’s, GCP and applicable regulatory requirements may be done at any time in future. In that case, the investigators will be provided with no less than one month official notification.

# After the trial

Completion of participant follow up at day 365 will not mark the end of trial related activities

**Cross over immunisation**

All the children who where allocated to the MSP 3 vaccine arm will be invited for cross over immunisation with Hepatitis B vaccine. This will only be possible after “data base freeze”, and the blind code opened to reveal the vaccine allocations. The sponsor will ensure that adequate doses of the control vaccine are available for this purpose.

# Publication and results dissemination

The principal investigator together with the AMANET clinical trials coordinator will be responsible for compiling all information relevant for production of the clinical study report. According to AMANET procedures, the clinical and statistical reports shall be written in the format of ICH GCP E3 and E9 guidelines.

This trial is being registered with the ATM Clinical Trials Registry at the South African Cochrane Centre, and AMANET is responsible for the registration.

It is the responsibility of the principal investigator to initiate and coordinate peer reviewed journal publication of the study results. The AMANET policy concerning publication stipulate:

- The investigator team shall be responsible for the dissemination of the scientific content of their work;
- Any publications arising from AMANET sponsored work must be submitted to AMANET for approval of content before being released to journals. A notification period of one month is given during which if no response is given, the investigator may go ahead. AMANET will not unreasonably withhold publication of study results.
- Each person listed as author must have participated actively in the conception, design, conduct, analysis and manuscript writing of the work. Such a person must be able to defend and explain the results by themselves.

# Appendices

Appendices to this protocol will include the following documents:

1. Participant Information sheet and informed Consent
2. AMANET DSMB Charter
3. Helsinki Declaration 2000 version
4. ICH GCP guidelines E 6 series
5. AMANET Trial Insurance policy cover
6. CNRFP vaccine trial SOP’s

**References**

1. Miller,L.H., Baruch,D.I., Marsh,K. & Doumbo,O.K. The pathogenic basis of malaria. Nature 415, 673-679 (2002)..
2. Chitnis,C.E. Molecular insights into receptors used by malaria parasites for erythrocyte invasion. Curr. Opin. Hematol. 8, 85-91 (2001).
3. Dvorak,J.A., Miller,L.H., Whitehouse,W.C. & Shiroishi,T. Invasion of erythrocytes by malaria merozoites. Science 187, 748-750 (1975).
4. Aikawa,M., Miller,L.H., Johnson,J. & Rabbege,J. Erythrocyte entry by malarial parasites. A moving junction between erythrocyte and parasite. J. Cell Biol. 77, 72-82 (1978).
5. Gysin J., Moisson P., Pereira da Silva L., and Druilhe PAntibodies from immune African donors with a protective effect in Plasmodium falciparum human infection are also able to control asexual blood forms of the parasite in Saimiri monkeys. Res Immunol 147:397-401. . 1996.
6. Oeuvray C., Bouharoun-Tayoun H., Gras-Masse H., et al. 1994. Merozoite surface protein-3: a malaria protein inducing antibodies that promote Plasmodium falciparum killing by cooperation with blood monocytes. Blood 84:1594-602.
7. Oeuvray, C., Bouharoun-Tayoun H., Grass-Masse H., et al . 1994. A novel merozoite surface antigen of Plasmodium falciparum (MSP3) identified by cellular-antibody cooperative mechanism antigenicity and biological activity of antibodies. Mem Inst Oswaldo Cruz 89:77-80.
8. Aribot G., Rogier C., Sarthou J. L. et al. 1996. Pattern of immunoglobulin isotype response to Plasmodium falciparum blood-stage antigens in individuals living in a holoendemic area of Senegal (Dielmo, west Africa). Am J Trop Med Hyg 54:449
9. Oeuvray C., Bouharoun-Tayoun H., Gras-Masse H., et al. 1994. Merozoite surface protein-3: a malaria protein inducing antibodies that promote Plasmodium falciparum killing by cooperation with blood monocytes. Blood 84:1594-602
10. Druilhe, P., Daubersies P., Patarapotikul J., et al . 1998. A primary malarial infection is composed of a very wide range of genetically diverse but related parasites. J Clin Invest 101:2008-16.
11. Aribot G., Rogier C., Sarthou J. L. et al. 1996. Pattern of immunoglobulin isotype response to Plasmodium falciparum blood-stage antigens in individuals living in a holoendemic area of Senegal (Dielmo, west Africa). Am J Trop Med Hyg 54:449-57
12. Badell, E., C. Oeuvray, A. Moreno, et al . 2000. Human Malaria in Immunocompromised Mice. An in vivo model to study defense mechanisms against plasmodium falciparum. J Exp Med 192:1653-1660.
13. Bouharoun-Tayoun H., Oeuvray C., Lunel F., and Druilhe P. 1995. Mechanisms underlying the monocyte-mediated antibody-dependent killing of Plasmodium falciparum asexual blood stages. J Exp Med 182:409-18.
14. Guiguemde, T. R., Coulibaly, N., Coulibaly, S. O., et al. 1997 [An outline of a method for estimating the calculated economic cost of malaria cases: its application to a rural area in Burkina Faso (Western Africa)]. Trop Med Int Health 2, 646-653.
15. Health Reports, Burkina Faso Ministry of Health, 1998.
16. Badell, E., C. Oeuvray, A. Moreno, et al . 2000. Human Malaria in Immunocompromised Mice. An in vivo model to study defense mechanisms against plasmodium falciparum. J Exp Med 192:1653-1660.
17. Gysin J., Moisson P., Pereira da Silva L., and Druilhe P. 1996. Antibodies from immune African donors with a protective effect in Plasmodium falciparum human infection are also able to control asexual blood forms of the parasite in Saimiri monkeys. Res Immunol 147:397-401.
18. Hisaeda H, Saul A, Reece JJ et al.. 2002 Merozoite surface protein 3 and protection against malaria in Aotus nancymai monkeys. J Infect Dis 1;185(5):657-64.
19. P. Druilhe, F. Spertini, G.P. Corradin,et al. 2002.Safety and Immunogenicity of the Merozoite-Surface-Protein-3 Antigen in Phase I Trials and Assessment of the Biological Effect of the Antibodies Induced. The Third MIM Pan-African Malaria Conference. Arusha, Tanzania
20. Snow, R.W., Craig,M., Deichmann,U. & Marsh,K. Estimating mortality, morbidity and disability due to malaria among Africa's non-pregnant population. Bull. World Health Organ 77, 624-640 (1999).
21. Alonso, P.L., Armstrong,J.R. & Lindsay,S.W. Malaria, bednets, and mortality. Lancet 338, 897 (1991).
22. D'Alessandro,U. et al. Mortality and morbidity from malaria in Gambian children after introduction of an impregnated bednet programme. Lancet 345, 479-483 (1995).
23. Nevill,C.G. et al. Insecticide-treated bednets reduce mortality and severe morbidity from malaria among children on the Kenyan coast. Trop. Med. Int. Health 1, 139-146 (1996).
24. Brooker,S. et al. Situation analysis of malaria in school-aged children in Kenya - what can be done? Parasitol. Today 16, 183-186 (2000).
25. Sachs,J. & Malaney,P. The economic and social burden of malaria. Nature 415, 680-685 (2002).
